# Effectiveness of Biological Surrogates for Predicting Patterns of Marine Biodiversity: A Global Meta-Analysis

**DOI:** 10.1371/journal.pone.0020141

**Published:** 2011-06-14

**Authors:** Camille Mellin, Steve Delean, Julian Caley, Graham Edgar, Mark Meekan, Roland Pitcher, Rachel Przeslawski, Alan Williams, Corey Bradshaw

**Affiliations:** 1 Australian Institute of Marine Science, Townsville, Queensland, Australia; 2 The Environment Institute and School of Earth and Environmental Sciences, University of Adelaide, Adelaide, South Australia, Australia; 3 Australian Commonwealth Scientific and Industrial Research Organisation, Marine and Atmospheric Research, Cleveland, Queensland, Australia; 4 Marine Research Laboratory, Tasmanian Aquaculture and Fisheries Institute, University of Tasmania, Taroona, Tasmania, Australia; 5 Geoscience Australia, Canberra, Australian Capital Territory, Australia; 6 South Australian Research and Development Institute, Henley Beach, South Australia, Australia; Argonne National Laboratory, United States of America

## Abstract

The use of biological surrogates as proxies for biodiversity patterns is gaining popularity, particularly in marine systems where field surveys can be expensive and species richness high. Yet, uncertainty regarding their applicability remains because of inconsistency of definitions, a lack of standard methods for estimating effectiveness, and variable spatial scales considered. We present a Bayesian meta-analysis of the effectiveness of biological surrogates in marine ecosystems. Surrogate effectiveness was defined both as the proportion of surrogacy tests where predictions based on surrogates were better than random (i.e., low probability of making a Type I error; *P*) and as the predictability of targets using surrogates (*R* ^2^). A total of 264 published surrogacy tests combined with prior probabilities elicited from eight international experts demonstrated that the habitat, spatial scale, type of surrogate and statistical method used all influenced surrogate effectiveness, at least according to either *P* or *R* ^2^. The type of surrogate used (higher-taxa, cross-taxa or subset taxa) was the best predictor of *P*, with the higher-taxa surrogates outperforming all others. The marine habitat was the best predictor of *R* ^2^, with particularly low predictability in tropical reefs. Surrogate effectiveness was greatest for higher-taxa surrogates at a <10-km spatial scale, in low-complexity marine habitats such as soft bottoms, and using multivariate-based methods. Comparisons with terrestrial studies in terms of the methods used to study surrogates revealed that marine applications still ignore some problems with several widely used statistical approaches to surrogacy. Our study provides a benchmark for the reliable use of biological surrogates in marine ecosystems, and highlights directions for future development of biological surrogates in predicting biodiversity.

## Introduction

*Biodiversity* is the term used to describe the collective variety of life from molecules to ecosystems, and from alleles to kingdoms. The diverse ways in which biodiversity can be defined has often impeded its precise use in conservation planning and policy development [1], [2]. Prioritizing areas for conservation does not, however, require a complete description of the biodiversity in areas of conservation concern, but can be based on relative measures of differences among them [3], [4]. Typically, estimation of these relative differences relies on the use of some simple estimator, a surrogate (e.g., the number of species in a particular taxon in a particular area) that is sufficiently related to the biodiversity parameter of interest, the target (e.g., the total number of species in that area [1], [5]). Surrogates of marine biodiversity patterns can be either physical [6] or biological. During recent decades, the latter have become increasingly necessary and useful in conservation science to bridge the gap between the scale of ecological observations and the scale of planning for conservation management [2], [7], [8], highlighting the need to understand clearly how well such surrogates perform under different conditions.

Interest in biological surrogates during the last decade has resulted in a growing number of studies about their effectiveness, in a variety of locations and at various spatial scales. It has also resulted in the definition of many types of biological surrogates and methods for their construction, partly because of recognized shortcomings of some well-established methods used to construct surrogates. For example, prioritizing habitats for conservation based on species richness only, observed or predicted, at particular sites (i.e. alpha diversity) might result in a selection of species-rich sites containing similar subsets of species. If so, rare species, or those only present in species-poor sites, could be excluded from protection [9]. To overcome such difficulties, many different methods have been developed, including those based on multivariate measures of biodiversity (i.e., derived from the matrix of site-specific species abundances [10]–[12]) or reserve-selection algorithms that maximize complementarity, such as the total number of species represented by a set of sites (e.g., [1]). These algorithms have recently been integrated into widely used conservation planning software packages such as Marxan [13], which are now used globally to address practical issues of reserve design. However, the extent to which surrogate effectiveness depends on the methods used and definitions employed has so far remained unexplored.

The need for effective biological surrogates is especially acute in the marine realm. A major impediment to area prioritization for marine conservation is the lack of information about the distribution of many marine species [10]. These gaps in our knowledge are mostly due to the large number of species that remain undescribed, difficulties in species identification, and the high costs of marine biodiversity surveys [14]–[16]. While a meta-analysis of the effectiveness of biological surrogates has been conducted in terrestrial ecosystems [17], this task remains to be done in the marine realm. Unless cost-effective biological surrogates are identified that can be used to prioritize areas for maximum conservation benefit, accelerating human impacts on most marine ecosystems could cause the decline and ultimately, the extinction of many marine species before they have been discovered.

We assess the effectiveness of biological surrogates as predictors of biodiversity in marine ecosystems using a Bayesian meta-analysis. Bayesian methods offer the unique opportunity to incorporate relevant prior knowledge explicitly into the analysis, i.e. a probability distribution of what is already known about a response variable [18]. Bayesian modelling techniques are well-adapted to ecological meta-analyses, where (*i*) the true number of independent studies are limited compared to the uncertainty and complexity of ecological systems, (*ii*) the results might be subject to publication bias [19] and (*iii*) expert knowledge acquired through field work or publication in gray literature is available to estimate priors. Recent interest in using expert knowledge in the elicitation of priors has led to the development of survey methods for this purpose [20], [21].

Here we test the hypotheses that spatial scale, habitat, surrogate type, and statistical approach can determine the effectiveness of biological surrogates. To accommodate the multiple definitions found in the literature, biological surrogates are hereafter defined in their widest sense and can include species in one genus, class, family or phylum, for which the biodiversity metrics that are compared to those of the target taxon can be either univariate (e.g., taxonomic richness, abundance, biomass) or multivariate (i.e., species presence/absence or abundance matrix). We defined surrogate effectiveness both as the proportion of tests where predictions based on surrogates performed ‘better’ than random (i.e., low probability of making a Type I error; *P*) and as the predictability of targets using surrogates (*R* ^2^). Our specific aims were to (*i*) review and classify the surrogates, methods and spatial scales considered so far in different marine habitats, (*ii*) test their effectiveness as predictors of biodiversity in a variety of habitats, at different spatial scales and using different definitions of surrogates and statistical methods to construct them, and (*iii*) formulate recommendations for the more reliable use of surrogates into the future, as they become ineluctable tools in conservation science. Our results also highlight directions for the further development of the application of biological surrogates in marine and other ecosystems.

## Methods

### Literature review and meta-data compilation

**Figure 1:**
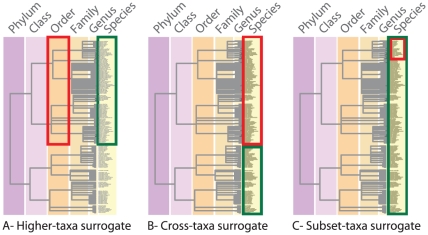
The different types of biological surrogates (red) and their targets (green). (A) Higher-taxa, where a taxon (or taxa) at a higher taxonomic level acts as surrogates for taxa at lower levels, (B) cross-taxa surrogates, where a taxon (or taxa) acts as a surrogate for another taxon (or taxa) at the same taxonomic level, and (C) subset-taxa surrogates, where a particular taxon (or taxa) acts as a surrogate for the entire target community. See Table S2 for referenced examples of each type of biological surrogate.

We conducted a meta-analysis of the peer-reviewed literature on biological surrogacy in marine ecosystems. Published studies testing biological surrogacy in marine ecosystems were identified using ISI's Web of Science® (www.isinet.com) and Elsevier's Science Direct® (www.sciencedirect.com) databases using the keywords ‘biodiversity’, ‘biological’, ‘diversity’, ‘indicator’, ‘proxy’, ‘surroga*’ and ‘tax*’. Each surrogacy test (i.e., sampling unit of the meta-analysis) was classified by the marine habitat studied (*Habitat*: soft bottom, temperate reef, or tropical coral reef), the spatial scale sampled, i.e. spatial extent of area over which samples were taken (*Scale*: <10, 10–100, >100 km), the type of surrogate used, defined by its relationship with the target (*Type*: higher-taxa, where a taxon acts as a surrogate for taxa at lower taxonomic levels; cross-taxa, where a taxon acts as a surrogate for another taxon at the same taxonomic level, or; subset-taxa surrogate, where a taxon acts as a surrogate for the entire target community; Figure 1) and the statistical approach used to construct the surrogate (*Stats*: spatial congruence of univariate biodiversity metrics; spatial congruence of multivariate biodiversity metrics; or representation, which uses site-selection algorithms to assess whether a network of areas selected to maximize the number of surrogate taxa captures a greater number of target taxa than expected by chance; Table 1). The sample size of each surrogacy test (*n*) was also recorded to account for different sampling intensities across studies.

**Table 1:** Statistical methods and biodiversity metrics used in marine biological surrogacy studies.

Method	Statistical index	Biodiversity metrics	Description of biodiversity metric	Test ID (examples)
▪ Congruence of univariate biodiversity metrics	▪ Spearman's rho or Pearson's r	▪ Taxonomic richness	▪ Number of taxa (e.g., species, genera)	1–3, 60–65, 98–101
		▪ Numerical Rarity	▪ Number of rare species (e.g., n≤2)	57, 59
		▪ Endemicity	▪ Number of endemic species (e.g., based on range extent)	102
		▪ Abundance	▪ Number of individuals (per unit area)	74, 218, 220–222
		▪ Biomass	▪ Mass per unit area	73, 75
▪ Congruence of multivariate biodiversity metrics	▪ Spearman's rho or Pearson's r between surrogate and target	▪ Incidence matrix	▪ Presence or absence of taxa (columns) at the different sites (rows)	4, 9–23
	▪ Bray-Curtis distance matrices	▪ Community composition	▪ Abundances of taxa (columns) at the different sites (rows)	76–97, 103–108
▪ Representation	▪ Site-selection algorithms	▪ Taxonomic richness	▪ Number of taxa (e.g., species, genera)	5, 7, 25–55
		▪ Numerical Rarity	▪ Number of rare species	6, 8
		▪ Occurrence of	▪ Taxa grouped according	149–162
		assemblages	to similarity in distribution	

Methods used are grouped into three categories: congruence of univariate biodiversity metrics assesses whether surrogate biodiversity is spatially correlated with target biodiversity; congruence of multivariate biodiversity metrics evaluates whether pairs of sites showing the highest similarity in surrogate assemblages also show the highest similarity in target assemblages, and; representation uses site-selection algorithms to assess whether a network of areas selected to maximize the number of surrogate taxa also maximises the number of target taxa and whether this number is greater than expected by chance. Test ID refers to Table S2.

From each surrogacy test, whether or not the null hypothesis was rejected (based on the authors' arbitrarily chosen threshold), and the coefficient of determination (*R* ^2^) of surrogate predictive power were collated. Overall surrogate effectiveness was then defined using both the probability of concluding that a surrogate performed better than random (*P*) and the surrogate predictive power (*R* ^2^). Ideally, surrogate effectiveness would be assessed as the likelihood of surrogate non-randomness based on bias-corrected maximum likelihoods for multi-model comparisons [22] or secondarily, as the probability of making a Type I error when concluding that surrogate predictions performed better than random. Such approaches could not be adopted here because most surrogacy studies have only reported an arbitrary probability threshold (e.g., *P*<0.05) when testing the null hypothesis that the surrogate did not perform better than random.

A central assumption of meta-analyses is that the literature reviewed is not subject to publication bias, which can arise if the probability an article is published depends on the strength and direction of its results [19]. We tested for publication bias using funnel plots of sample size against effect size, i.e., the number of tests in each 0.05-class of *R* ^2^ (not shown; [23]) combined with a rank correlation test between standardized sample size and effect size.

### Elicitation of priors using expert knowledge

Eight international experts in marine biological surrogacy answered an online survey (http://www.adelaide.edu.au/environment/mbp/survey/02.html) on the effectiveness of biological surrogates in marine ecosystems. They were asked to estimate the likelihood (*P*) of a surrogate being effective in predicting a target, and the proportion of variance explained (*R* ^2^) by the surrogate for the different habitats, scales, types of surrogates and statistical approaches defined above. In each situation, experts were asked to classify *P* and *R* ^2^ into one of five categories (0.0–0.2; 0.2–0.4; 0.4–0.6; 0.6–0.8; 0.8–1). Additionally, they were asked to indicate their confidence in the classification using the same categories. We translated these classes into a categorical estimate from ‘very low/small’ to ‘very high/large’ to facilitate interpretation by the experts [20]. Their type and level of expertise were assessed using questions on their research activities and proportion of time allocated to the study of marine biological surrogates. The effect of their level of expertise and how it was acquired on their confidence when answering these questions was investigated with a multivariate analysis of variance using a Bray-Curtis distance matrix and 100 permutations (NPMANOVA; [24]). The statistical units were experts, and multivariate responses were their mean confidence score when answering questions relating to the *Habitat*, *Scale*, *Type*, or *Stats* factors. Predictors were the descriptors of their expertise (Table S1).

### Bayesian model fitting

Bayesian hierarchical (i.e., multilevel) models of surrogate effectiveness, successively defined as *P* and *R* ^2^, were implemented to assess the influence of the factors defined above on surrogate effectiveness. For each response variable, covariates included each of the factors *Habitat*, *Scale*, *Type*, *Stats* coded as dummy variables, or *n*, in a separate model. The hierarchical term *Study* was added to account for the non-independence of multiple tests within the same study. The resulting model formulation is given by:
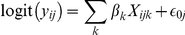


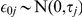
where *y_ij_* is the surrogate effectiveness for each test *i* of study *j*, *β*_k_ is the coefficient associated with the *k* ^th^ (dummy) covariate *X_ijk_*, *ε_0j_* is the effect for the *j* ^th^ study and *τ_j_* is gamma-distributed (*a* = 0.001, *b* = 0.001). The binary *P* response variable was modeled using a binomial density function. The continuous *R* ^2^ response variable, which is restricted to the interval [0, 1], was modeled using a beta density function (see code in Text S1; [25]). The logit link function was used for both response variables. Both uninformative priors *β*_k_∼N(0, 1000) and informative priors *β*_k_∼N(*μ*_k_, *σ*_k_^2^), where *μ*_k_ and *σ*_k_^2^ were estimated based on expert opinion, were considered. Therefore, for each response variable and each covariate, three models were considered with (*i*) covariate effect only and uninformative priors (*ii*) covariate and hierarchical effects and uninformative priors and (*iii*) covariate and hierarchical effects and informative priors. Model performance was assessed using the deviance information criterion (DIC) [26]. Model parameters were estimated using Markov chain Monte Carlo (MCMC) and Gibbs sampling. To ensure model convergence, models were run for 50,000 iterations with a 2,000 iteration burn-in period and every 12^th^ observation was retained to control for any potential autocorrelation; the remaining 4000 values of each parameter were retained to generate the posterior distributions of the parameters. Model fitting was done using WinBUGS 1.4 [27] and the R2WinBUGS [28] package for R 2.9.2 [29].

## Results

### Literature review and meta-data compilation

Peer-reviewed and published literature on biological surrogacy in marine ecosystems included 20 studies presenting 264 biological surrogacy tests (Table S2; Text S2). Of these 264 tests, 138 were for soft bottom habitats (all in temperate regions), 71 were for temperate reefs and 55 for coral reefs (see Table 2 for cross-factor tabulations). These surrogacy tests were distributed globally, with 10 tests from the Arctic, 114 from Australia, 55 from the Indo-Pacific (including Indonesia, Madagascar, the Philippines and Papua New Guinea), 59 from the Mediterranean Sea and 26 from northern Europe. For higher-taxa surrogates, there were a variable number of taxonomic steps between the surrogate and the target, and the surrogate predictive power decreased as the number of taxonomic steps between the surrogate and the target increased (Table 3). We found no evidence of any publication bias in the surrogate predictive power: i.e., no consistent trend between the number of published articles and surrogate effectiveness (Spearman's *ρ* = −0.20; *P* = 0.38).

**Table 2:** Cross-tabulations of the number of tests for each combination of factor levels.

		Habitat			Type			Scale			Method		
		TR	TE	SO	CT	HT	ST	L	M	S	UC	MC	RP
Habitat	TR	55			55	0	0	2	53	0	5	16	34
	TE		71		20	36	15	29	27	15	24	39	8
	SO			138	27	92	19	16	80	42	32	84	22
Type	CT				102			22	65	15	21	44	37
	HT					128		14	77	37	20	86	22
	ST						34	11	18	5	20	9	5
Scale	L							47			19	28	0
	M								160		24	80	56
	S									57	18	31	8
Method	UC										61		
	MC											139	
	RP												64

The diagonal indicates the total number of tests in each factor level including, for *Habitat*, TR: tropical reefs, TE: temperate reefs, SO: soft bottoms; for *Type*, CT: cross-taxa surrogate, HT: higher-taxa surrogate, ST: subset-taxa surrogate; for *Scale*, L: >100 km, M: 10–100 km, S: <10 km; and for *Method*, UC: univariate congruence, MC: multivariate congruence, RP: representation.

**Table 3:** Higher-taxa surrogate predictive power (*R* ^2^) as a function of the number of taxonomic steps between the surrogate and the target.

Nb steps	Surrogate	Target	*R* ^2^ mean	*R* ^2^ sd	*n*	mean *R* ^2^	*n*_tot_
1	class	order	0.19	0.08	5	0.61	36
	order	family	0.47	0.06	6		
	family	genus	0.87	0.08	6		
	genus	species	0.91	0.09	19		
2	class	family	0.11	0.09	5	0.43	34
	order	genus	0.39	0.1	6		
	family	species	0.78	0.19	23		
3	class	genus	0.11	0.09	5	0.29	23
	order	species	0.48	0.25	18		
4	class	species	0.25	0.18	10	0.25	10

With *sd* = standard deviation, *n* = number of tests, *n*_tot_ = total number of tests.

### Elicitation of priors using expert knowledge

Most experts answered all questions within 15 to 30 minutes (Table S1) and left less than 10% of the questions (24 of 288) unanswered. Expert confidence averaged 58±13% (mean ± standard deviation), corresponding to ‘fairly confident’ according to the survey terminology. Mean expert confidence was influenced by their experience in statistical analysis only (NPMANOVA *R* ^2^ = 0.30; *P* = 0.040).

**Figure 2:**
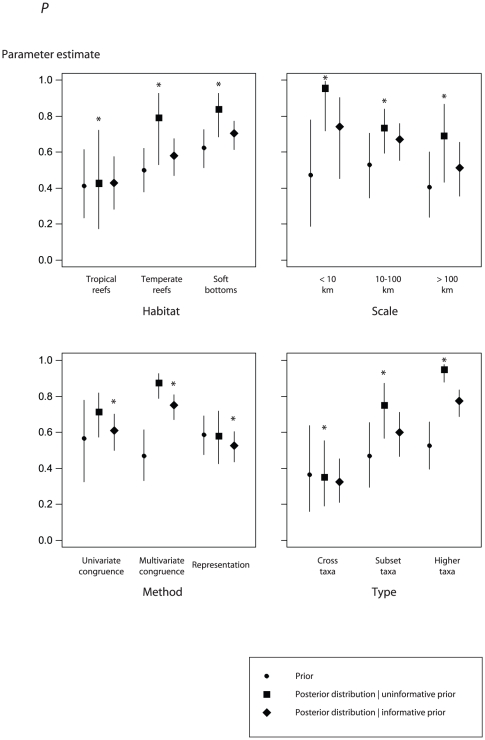
Surrogate effectiveness defined by *P*, the proportion of tests concluding that surrogate predictions were non-random. Prior distribution (circles), posterior distribution given an uninformative prior (analogous to the likelihood; squares), and posterior distribution given an informative prior (diamonds). Error bars depict the standard deviation of the prior or posterior. Asterisks indicate the best model according to the deviance information criterion. Factors include the marine habitat (*Habitat*), spatial scale (*Scale*), the statistical method used to assess surrogate performance (*Method*) and the type of surrogate (*Type*).

**Figure 3:**
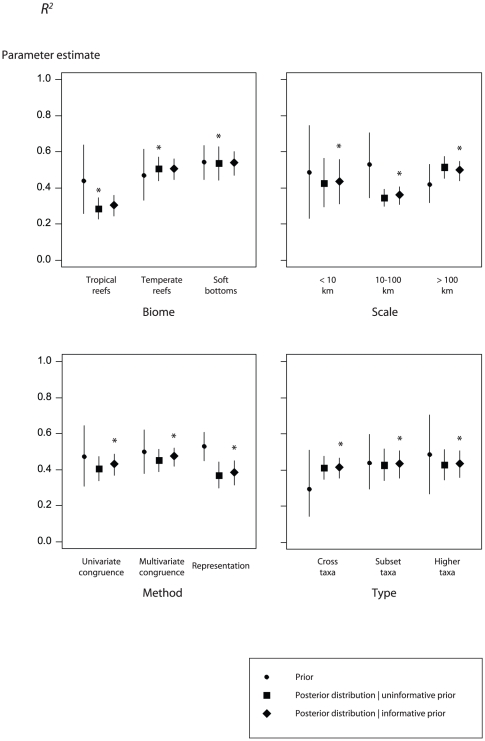
Surrogate effectiveness defined by *R^2^*, the predictability of targets using surrogates. Prior distribution (circles), posterior distribution given an uninformative prior (analogous to the likelihood; squares) and posterior distribution given an informative prior (diamonds). Error bars depict the standard deviation of the prior or posterior. Asterisks indicate the best model according to the deviance information criterion. Factors include the marine habitat (*Habitat*), spatial scale (*Scale*), the statistical method used to assess surrogate performance (*Method*) and the type of surrogate (*Type*).

Experts' ranking of *P* was the highest in soft bottom habitats, at a 10–100 km spatial scale, using representation-based statistical methods and a higher-taxa surrogate (Figure 2: circles). Experts' ranking of *R* ^2^ was in agreement with that of *P*, although differences among factor levels were less pronounced (Figure 3: circles).

### Bayesian model fitting

**Figure 4:**
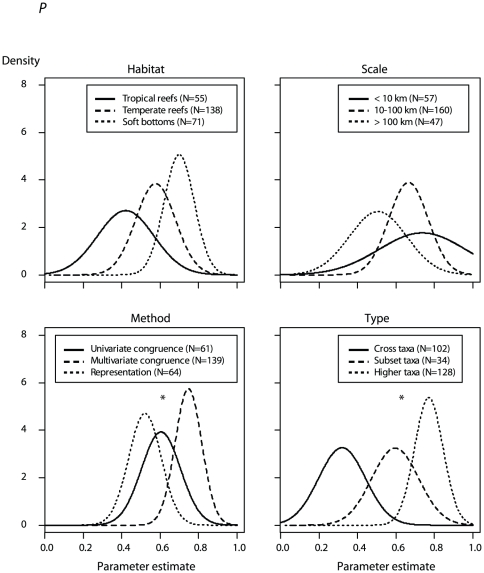
Posterior distributions (given an informative prior) of surrogate effectiveness defined as *P*. Posterior distributions of *P* (i.e. the proportion of tests concluding that surrogate predictions are non-random) are given according to the marine habitat (*Habitat*), spatial scale (*Scale*), the statistical method used to assess surrogate performance (*Method*) and the type of surrogate (*Type*). Asterisks indicate models outperforming the null model. A Gaussian distribution with the mean and standard deviation of the posterior distribution was used to approximate posterior distributions.

**Figure 5:**
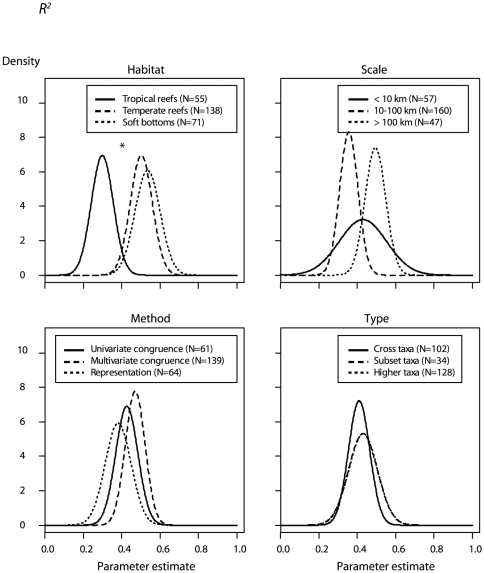
Posterior distributions (given an informative prior) of surrogate effectiveness defined as *R* ^2^. Posterior distributions of *R* ^2^ (i.e. the surrogate predictive power) are given according to the marine habitat (*Habitat*), spatial scale (*Scale*), the statistical method used to assess surrogate performance (*Method*) and the type of surrogate (*Type*). Asterisks indicate models outperforming the null model. A Gaussian distribution with the mean and standard deviation of the posterior distribution was used to approximate posterior distributions.

The *Type* model of the *P* response variable was top-ranked according to the deviance information criterion (DIC), with higher- taxa surrogates, and subset-taxa surrogates to a lesser extent, both predicting higher *P* than cross-taxa surrogates (odds ratio = 60.1 and 10.0, respectively; Table 4; Figure 4). The type of habitat best explained *R* ^2^, with both soft bottoms and temperate reefs performing better than tropical reef (odds ratio = 7.3 and 6.4, respectively; Table 4; Figure 5). All factors were important predictors of *P*, but only the type of habitat and the sample size models were ranked higher than the null model for the *R* ^2^ response (Table 4). For both response variables, hierarchical models that incorporated a *Study* effect accounting for the non-independence of tests within the same study ranked higher than the covariate-effects-only models (Table 4).

**Table 4:** Deviance information criterion (DIC) for models of surrogate effectiveness defined as the proportion of tests (P) concluding that surrogate predictions are non-random and as the surrogate predictive power (R^2^).

Response	Model	Model type	Priors	DIC
P	type	mixed		207.31
	type	mixed	informative	208.96
	method	mixed	informative	212.66
	method	mixed		212.76
	scale	mixed		217.44
	*n*	mixed		218.08
	habitat	mixed		218.19
	null	mixed		218.26
	habitat	mixed	informative	218.47
	scale	mixed	informative	218.59
	type	fixed		221.51
	method	fixed		248.06
	habitat	fixed		253.63
	scale	fixed		266.84
	null	fixed		269.33
	*n*	fixed		270.99
R^2^	habitat	mixed		−74.55
	habitat	mixed	informative	−74.11
	*n*	mixed		−73.14
	null	mixed		−71.98
	type	mixed	informative	−69.39
	type	mixed		−69
	method	mixed	informative	−68.06
	method	mixed		−68
	scale	mixed	informative	−66.41
	scale	mixed		−65.83
	scale	fixed		−62.25
	habitat	fixed		−61.61
	*n*	fixed		−53.35
	method	fixed		−52.98
	null	fixed		−46.28
	type	fixed		−43.81

Factors include the type of surrogate (type), the statistical method used to assess surrogate performance (method), the marine habitat (habitat), the spatial scale (scale) and the sample size (n). Models are ranked by increasing DIC.

Models incorporating an informative prior were ranked higher than those incorporating an uninformative prior for the *Method* model predicting *P* (Figure 2: diamonds; Table 4), and for the *Type*, *Method* and *Scale* models predicting *R* ^2^ (Figure 3: diamonds; Table 4). However, differences in DIC were generally small (i.e., ΔDIC<1, except for the *Scale* and *Type* models of *P*), indicating that models with uninformative or informative priors provided an approximately equal description of the data and were essentially undistinguishable. For the *Scale* and *Type* models of *P*, models with uninformative priors performed better than those with informative priors (Figure 2: squares). In the first case, priors based on expert opinion did not capture the pattern revealed by the literature, i.e., a decrease in surrogate effectiveness as spatial scale increases. In the second case, the ranking of factor levels according to expert opinion was in agreement with the meta-data; however, informative priors did not improve the model, as a consequence of their diffuse distribution and a precise estimate of the likelihood based on the meta-data only. The same situation was observed for the *Habitat* model of *R^2^* (Figure 3: squares).

Convergence was successfully achieved for all models, although we could not fit any model that included interactions because of missing cross-factor combinations. Models including additive effects of all covariates could not be fitted either; adding all terms aliased the random effect as a result of the low level of replication of the different cross-factor combinations across studies.

## Discussion

### Drivers of surrogate effectiveness

As a first attempt to collate information on biological surrogate effectiveness in marine ecosystems, our study highlighted overall that in most situations, surrogate effectiveness was typically lower than generally assumed. Even with a relatively low expectation of the relationship between surrogate and target biodiversity (i.e., non-random), in most situations more than one third of all studies found no evidence for such a relationship. Moreover, even when there was strong evidence for a relationship between surrogate and target biodiversity, the predictability of such a relationship was nevertheless often weak (e.g., at a spatial scale of 10–100 km; *P* = 0.66±0.10 and *R^2^* = 0.35±0.05). This clearly highlights the need to understand why some surrogates might not be appropriate in certain situations and to formulate precise recommendations for a more reliable use of biological surrogates in future studies of marine biodiversity.

By combining expert knowledge and published literature on surrogate effectiveness, we showed that the spatial scale, habitat, type of surrogate and method used for its construction all influenced surrogate effectiveness, according to at least one of the two measures of effectiveness considered. The type of surrogate used was the strongest determinant of *P*, with higher-taxa surrogates predicting higher *P* than all other types. This greater effectiveness of higher-taxa surrogates might be intuitively expected because of the hierarchical relationship among taxonomic levels [30] where the probability of observing a high number of genera increases with the number of species observed. However even though expected, this result is demonstrated here for the first time and warrants further development of higher-taxa surrogates, once one guards against a number of potential pitfalls. First, the rate of spatial variation in biodiversity metrics (*β* diversity) declines with decreasing taxonomic resolution [31], so inter-site differences in species richness or composition might not be detected by such higher-taxonomic level surrogates. Secondly, we advise caution when comparing the effectiveness of higher-taxa surrogates across taxonomic groups. Indeed, subdivisions of taxa and their rationale for classification into various taxonomic levels is inconsistent across groups because current taxonomic classifications result from a heterogeneous mixture of various historical and contemporary views [32]. Therefore, we contend that higher-taxa approaches can provide valuable surrogates only at a scale where they reflect species-level patterns of *β* diversity, and as long as the inherent uncertainty of taxonomic classifications tempers conclusions.

Our results indicate that surrogates based on representation were less effective than those based on spatial congruence of univariate or multivariate biodiversity metrics. Representation-based methods, which consist of selecting a set of sites based on a surrogate and summing the representation of the target within the selected set, suffer from a number of flaws previously highlighted in studies of surrogacy in terrestrial ecosystems. Site-selection algorithms return one solution for reserve design from a large number of potentials which might not be optimal because they cannot assess the overall pattern of representation [33]. Moreover, representation-based surrogates are designed to perform better than the random addition of sites, but they are rarely compared to the optimum selection of sites, derived by selecting sites on the basis of the target instead of the surrogate – an approach that has been developed in terrestrial ecosystems [34]. Such methods have yet to be applied in marine ecosystems but appear worthy of exploration in this realm. Lastly, representation-based methods can and should be used with biodiversity metrics other than just taxonomic richness. The number of taxa observed at a given place depends strongly on sampling effort [35], and this might influence the relationship between surrogate and target species richness.

Surrogate effectiveness also decreased with increasing spatial scale. Most studies conducted at spatial scales >100 km assumed homogeneity of the study system at finer scales. Such an assumption might be incorrect if different biogeographic sub-regions or distinct evolutionary histories are included [36], [37], or if different combinations of habitats are represented among areas. Indeed, surrogate performance varies among areas displaying regional variation of species distributions [8], [38] and patterns of ‘local endemism’ (i.e. fine-scale patterns in species-habitat associations; [37]). Despite some evidence of the importance of spatial scale on surrogate effectiveness, such effects are still largely unknown.

Surrogate effectiveness varied among habitats and was lowest for tropical coral reefs. The high biodiversity and functional complexity of coral reefs might have been responsible for weaker relationships between surrogates and their target taxa. Indeed, both theoretical and experimental evidence suggest that species diversity, which is exceptionally high in tropical coral reefs [39], [40], is strongly correlated to functional diversity [41], [42]. This strong coupling could reflect a high partitioning of resources among component species and low overlap in their functional traits [42]. Thus in tropical reefs, a large number of functional groups often results in more complex food webs. These results corroborate the idea that high species and functional diversity characterizing tropical coral reefs enhances ecosystem complexity, thus weakening predictive and surrogacy relationships between taxa.

### Value of Bayesian mixed-effect models in meta-analyses

Using Bayesian hierarchical models in a meta-analysis of surrogate effectiveness allowed us to account for correlation among multiple statistical tests within studies. All hierarchical models ranked higher than the analogous non-hierarchical models, showing the relevance of accounting for such correlation, and suggesting that studies neglecting them should be interpreted with caution (e.g., [17]). Multi-level hierarchical models have been extensively used in socio-economic and medical research, for example, to model the relationship among households of a same city [43], but such models have only recently received interest in ecology (e.g., [44], [45]). A frequentist framework could alternatively be used to model such within-group correlations; however, when the number of groups is small or the multi-level design is complicated, there might not be enough information to estimate variance components precisely, whereas a Bayesian approach would average over the uncertainty in all parameters in the model [43].

Incorporating an informative prior in our models proved valuable in a few cases where estimations of the likelihood based on meta-data only were relatively imprecise, or differences among factor levels were small, such as in the *Method* model of *P*, or in the *Scale*, *Method* or *Type* models of *R* ^2^. In other cases, models using informative priors based on expert opinion and those using an uninformative prior were essentially undistinguishable. This might reflect a relatively diffuse distribution of the prior with respect to that of the estimate based on the meta-data. According to the terminology used by Kuhnert et al (2010), we used a direct-elicitation method, conducted remotely through an online survey. This has the advantage of eliciting opinion from multiple international experts when resource constraints prevent face-to-face interviews [20]. However, the drawback is that no Delphi process could be used, i.e., the process by which mutual feedback among experts promotes the convergence of their opinion to reach a consensus, or where the elicitor can provide immediate feedback to the expert in one-to-one style survey. Whether or not using the Delphi approach, expert opinion can still be prone to biases that can emerge from different sources (see Table 3 in Kuhnert et al. 2010). Among those sources, the linguistic uncertainty (inducing a *cognitive bias*, i.e. misunderstanding of what is required) [21] is probably the most accessible to the elicitor. In our survey, measures were taken to reduce the linguistic uncertainty and included the assistance of a social scientist to design the survey, as well as a series of dry tests and feed-back loops among research group colleagues (Mellin C, unpubl. data.).

### Recommendations for the use of biological surrogates

Despite an exhaustive literature search, a careful examination of the metadata revealed that specific surrogate and target groups were not uniformly distributed across spatial scales and habitats. For example, all studies targeting arthropods were done at a spatial scale >100 km (Table S2). We found that the lowest surrogate effectiveness, observed in tropical reefs, was always associated with tests examining corals. Although we could not fit any model that included interactions between taxon and spatial scale (or habitat) because of missing cross-factor combinations, we contend that associations between a specific taxon and a spatial scale (or habitat) did not bias overall results across all taxa considered (total of 16). However, these associations still reflect ecologists' expectations as to the spatial scales or habitats where surrogates should be the most effective, and can in turn be analysed qualitatively to revisit these expectations and inform the sampling design of future studies. Likewise, studies targeting arthropods could possibly benefit from the consideration of a spatial scale <10 km, whereas surrogate effectiveness in coral reefs could be higher when considering taxa other than corals.

We expect surrogate effectiveness to be the greatest for higher-taxa surrogates at a <10-km spatial scale, in low-complexity marine ecosystems such as soft bottoms, and using multivariate-based methods. In addition, surrogate taxa should ideally have a broad distribution across different environments [2] but also incorporate many species with restricted distributions [14], [33], [46], [47], be easy and cost-effective to identify and survey [47], and be amenable to survey at multiple spatial scales [48]. A lack of spatial consistency in surrogacy due, for example, to regional patterns in species distributions, might make indicator groups perform differently among areas [47].

While often used in combination and compared, the different statistical methods applied to construct the surrogate serve different purposes. One third of the studies examined here used a combination of methods based on spatial congruence and representation to answer the same question, despite the two approaches addressing different issues. While congruence-based methods (uni- or multivariate) are informative for prediction, they cannot inform conservation planning efficiency. Ensuring the cost-effectiveness of conservation efforts requires maximizing biodiversity (or endemism) included in a minimum number of sites [7], [49], [50]. This can only be achieved using representation-based methods such as reserve-selection algorithms. Therefore, where the goal of a study is the prediction of biodiversity patterns, surrogacy methods based on spatial congruence should be used; where conservation planning is the goal, surrogacy methods based on representation are applicable. However, such a distinction between the utility of these different approaches to biological surrogates is rarely apparent in the published literature. Future investigation of biological surrogacy will benefit from careful choice and specification of methods depending on whether the goal of the study is prediction or planning.

Independent of the ecosystems in which they are applied, the reliability of biological surrogates can be improved in the following ways: (*i*) Surrogates should be selected based on the target taxa of interest, with an awareness of the limits imposed by that selection. Higher-taxa surrogates can be appropriate (and often effective) when identification of target taxa to species is expensive, while cross-taxa surrogates are appropriate (but rarely effective) when the target is difficult to census. (*ii*) The objective of using a particular surrogate needs to be explicit, and the method used for its construction must be matched to this objective to maximize its effectiveness. If the surrogate is to be used for designing networks of protected areas, only representation-based methods are appropriate, whereas congruence-based methods should be used to predict patterns of biodiversity where data are scarce or unavailable. (*iii*) The costs of sampling surrogates need to be evaluated to optimize their efficiency. To be efficient, a surrogate must be considerably less expensive and time-consuming than sampling the target [2]. There is, however, currently little information available regarding the costs of monitoring biological surrogates in marine ecosystems (but see [51] for the case of tropical forests). (*iv*) Finally, the temporal stability of surrogate effectiveness needs to be tested and not just assumed. Most biological surrogacy studies have attempted to evaluate surrogate effectiveness across space only, pooling samples among times, without assessing the temporal robustness of the surrogate-target relationship (but see [52]). Doing so will be particularly useful in the context of monitoring the species-specific responses to global change. By advancing knowledge in these four areas, a better understanding of the properties of surrogates and how to deploy them most effectively and efficiently will be gained. Bridging these knowledge gaps is crucial as biological surrogates are becoming an increasingly important tool for conservation planning.

## Supporting Information

Table S1 Results obtained for the respondents of the online survey available at http://www.adelaide.edu.au/environment/mbp/survey/02.html.
(DOC)

Table S2 Meta-data and references of literature reviewed.
(DOC)

Text S1 WinBUGS code for Bayesian modelling.
(DOC)

Text S2 Literature reviewed.
(DOC)
