# Supplementary material for: Effectiveness of Biological Surrogates for Predicting Patterns of Marine Biodiversity: A Global Meta-Analysis
Source: PLoS One. 2011 Jun 14;6(6):e20141. doi: 10.1371/journal.pone.0020141 (PMC3114784; doi:10.1371/journal.pone.0020141)
Supplement: Table S1 — Results obtained for the respondents of the online survey available at http://www.adelaide.edu.au/environment/mbp/survey/02.html. (DOC) [file pone.0020141.s001.doc]

**Effectiveness of Biological Surrogates for Predicting Patterns of Marine Biodiversity : a Global Meta-Analysis**

Mellin et al. – Supporting Information

**Table S1** Results obtained for the respondents of the online survey available at <http://www.adelaide.edu.au/environment/mbp/survey/02.html>.

Table S1 Results obtained for the eight respondents of the online survey, with Habitat_1: coral reefs, 2: temperate reefs, 3: soft bottom; Type_1: higher-taxa, 2: cross-taxa, 3: subset-taxa; Scale_1: <10 km, 2: 10-100 km, 3: > 100 km; Method_1: congruence in univariate biodiversity metrics, 2: congruence in multivariate biodiversity metrics, 3: representation. P = probability, R² = coefficient of determination, conf = level of confidence.

|  |  |  |  |  |  | Expert |  |  |  |
| --- | --- | --- | --- | --- | --- | --- | --- | --- | --- |
|  |  | E1 | E2 | E3 | E4 | E5 | E6 | E7 | E8 |
| Expert typology | |  |  |  |  |  |  |  |  |
|  | Field work | 1 | 1 | 1 | 1 | 1 | 1 | 1 | 1 |
|  | Literature | 1 | 0 | 1 | 1 | 1 | 1 | 1 | 0 |
|  | Statistical analyses | 1 | 1 | 0 | 0 | 1 | 1 | 0 | 1 |
|  | Modelling | 0 | 0 | 0 | 0 | 0 | 1 | 0 | 0 |
|  | Other (conservation planning) | 0 | 0 | 0 | 0 | 1 | 0 | 0 | 0 |
|  | Temperate marine | 1 | 1 | 0 | 1 | 1 | 0 | 1 | 1 |
|  | Tropical marine | 0 | 0 | 1 | 1 | 0 | 1 | 1 | 0 |
|  | Reefs or rocky shores | 1 | 1 | 0 | 1 | 1 | 0 | 0 | 0 |
|  | Soft bottoms | 0 | 1 | 1 | 1 | 1 | 0 | 0 | 0 |
|  | Freshwater | 0 | 0 | 0 | 1 | 0 | 0 | 0 | 0 |
|  | Terrestrial | 0 | 0 | 0 | 1 | 0 | 0 | 0 | 0 |
|  | Other (estuaries) | 0 | 0 | 0 | 0 | 1 | 0 | 0 | 0 |
|  | Number of years | >10 | 5-Oct | >10 | >10 | 5-Oct | 5-Oct | 1-May | >10 |
|  | Involvement over the last years | 1 | 1 | 1 | 1 | 1 | 1 | 1 | 0 |
|  | Percentage of research time | <25 | 25-50 | <25 | 25-50 | 25-50 | <25 | <25 | 0 |
|  | Regression | 0 | 0 | 0 | 1 | 1 | 1 | 0 | 1 |
|  | GLM and GAM | 1 | 0 | 0 | 1 | 0 | 0 | 0 | 0 |
|  | Ordination | 0 | 1 | 1 | 1 | 1 | 0 | 1 | 1 |
|  | Site-selection algorithms | 0 | 0 | 0 | 1 | 1 | 1 | 0 | 1 |
|  | Other (GIS and spatial analysis) | 0 | 0 | 0 | 1 | 0 | 0 | 0 | 0 |
|  |  |  |  |  |  |  |  |  |  |
| Expert opinion | |  |  |  |  |  |  |  |  |
|  | Habitat_1_P | 0.5 | 0.7 | 0.1 | 0.3 | 0.5 | 0.1 | 0.5 | 0.5 |
|  | Habitat_1_R^2^ | 0.5 | 0.5 | 0.1 | 0.3 | 0.5 | 0.1 | 0.3 | 0.3 |
|  | Habitat_1_conf | 0.5 | 0.7 | 0.7 | 0.5 | 0.7 | 0.7 | 0.5 | 0.5 |
|  | Habitat_2_P | 0.7 | 0.3 | 0.7 | 0.5 | 0.5 | 0.3 | NA | 0.3 |
|  | Habitat_2_R^2^ | 0.7 | 0.5 | 0.3 | 0.5 | 0.5 | 0.3 | NA | 0.3 |
|  | Habitat_2_conf | 0.7 | 0.7 | 0.7 | 0.5 | 0.7 | 0.7 | NA | 0.5 |
|  | Habitat_3_P | 0.7 | 0.5 | 0.5 | 0.7 | 0.5 | 0.5 | 0.5 | 0.3 |
|  | Habitat_3_R^2^ | 0.7 | 0.5 | 0.5 | 0.7 | 0.5 | 0.5 | 0.5 | 0.1 |
|  | Habitat_3_conf | 0.7 | 0.5 | 0.7 | 0.7 | 0.7 | 0.7 | 0.3 | 0.5 |
|  |  |  |  |  |  |  |  |  |  |
|  | Type_1_P | NA | 0.3 | 0.7 | 0.7 | 0.3 | 0.3 | 0.3 | 0.3 |
|  | Type_1_R^2^ | NA | 0.7 | 0.5 | 0.7 | 0.3 | 0.3 | 0.3 | 0.3 |
|  | Type_1_conf | NA | 0.5 | 0.5 | 0.7 | 0.7 | 0.7 | 0.5 | 0.5 |
|  | Type_2_P | 0.5 | 0.5 | 0.5 | 0.7 | 0.3 | NA | 0.5 | 0.5 |
|  | Type_2_R^2^ | 0.5 | 0.5 | 0.5 | 0.7 | 0.3 | NA | 0.5 | 0.3 |
|  | Type_2_conf | 0.5 | 0.5 | 0.5 | 0.7 | 0.7 | 0.1 | 0.7 | 0.5 |
|  | Type_3_P | NA | 0.7 | 0.7 | 0.5 | 0.7 | NA | NA | 0.5 |
|  | Type_3_R^2^ | NA | 0.5 | 0.7 | 0.5 | 0.5 | NA | NA | 0.5 |
|  | Type_3_conf | NA | 0.5 | 0.7 | 0.5 | 0.7 | 0.1 | NA | 0.5 |
|  |  |  |  |  |  |  |  |  |  |
|  | Scale_1_P | 0.5 | 0.5 | 0.5 | 0.3 | 0.5 | 0.5 | 0.1 | 0.5 |
|  | Scale_1_R^2^ | 0.5 | 0.3 | 0.5 | 0.3 | 0.5 | 0.5 | 0.3 | 0.5 |
|  | Scale_1_conf | 0.5 | 0.5 | 0.5 | 0.5 | 0.5 | 0.5 | 0.5 | 0.5 |
|  | Scale_2_P | 0.7 | 0.5 | 0.7 | 0.7 | 0.5 | 0.3 | NA | 0.3 |
|  | Scale_2_R^2^ | 0.7 | 0.5 | 0.7 | 0.7 | 0.5 | 0.3 | NA | 0.3 |
|  | Scale_2_conf | 0.7 | 0.5 | 0.5 | 0.7 | 0.7 | 0.7 | NA | 0.5 |
|  | Scale_3_P | 0.7 | 0.3 | 0.7 | 0.9 | 0.3 | 0.1 | 0.5 | 0.3 |
|  | Scale_3_R^2^ | 0.7 | 0.7 | 0.7 | 0.7 | 0.3 | 0.1 | 0.5 | 0.3 |
|  | Scale_3_conf | 0.7 | 0.5 | 0.7 | 0.7 | 0.5 | 0.7 | 0.7 | 0.5 |
|  |  |  |  |  |  |  |  |  |  |
|  | Method_1_P | 0.7 | 0.9 | 0.7 | 0.5 | 0.5 | 0.5 | 0.3 | 0.3 |
|  | Method_1_R^2^ | 0.7 | 0.3 | 0.7 | 0.5 | 0.5 | 0.5 | 0.3 | 0.3 |
|  | Method_1_conf | 0.7 | 0.7 | 0.5 | 0.7 | 0.7 | 0.7 | 0.3 | 0.5 |
|  | Method_2_P | 0.3 | 0.3 | NA | 0.7 | 0.5 | 0.5 | 0.5 | 0.5 |
|  | Method_2_R^2^ | NA | 0.5 | 0.5 | 0.7 | 0.5 | 0.5 | 0.5 | 0.3 |
|  | Method_2_conf | 0.3 | 0.7 | 0.5 | 0.5 | 0.7 | 0.7 | 0.5 | 0.5 |
|  | Method_3_P | 0.5 | 0.7 | 0.5 | 0.7 | 0.5 | 0.7 | NA | 0.5 |
|  | Method_3_R^2^ | 0.5 | 0.5 | 0.5 | 0.7 | 0.5 | 0.5 | NA | 0.5 |
|  | Method_3_conf | 0.5 | 0.5 | 0.5 | 0.5 | 0.7 | 0.7 | NA | 0.5 |
|  |  |  |  |  |  |  |  |  |  |
| Expert involvement in the survey | | | |  |  |  |  |  |  |
|  | Acknowledgement desired | no | yes | yes | yes | yes | yes | no | yes |
|  | Time required (min) | 15-30 | 15-30 | 15-30 | <15 | 15-30 | 15-30 | 15-30 | 15-30 |
