# Supplementary material for: Effectiveness of Biological Surrogates for Predicting Patterns of Marine Biodiversity: A Global Meta-Analysis
Source: PLoS One. 2011 Jun 14;6(6):e20141. doi: 10.1371/journal.pone.0020141 (PMC3114784; doi:10.1371/journal.pone.0020141)
Supplement: Table S2 — Meta-data and references of literature reviewed. (DOC) [file pone.0020141.s002.doc]

**Effectiveness of Biological Surrogates for Predicting Patterns of Marine Biodiversity : a Global Meta-Analysis**

Mellin et al. – Supporting Information

**Table S2** Meta-data and references of literature reviewed.

Table S2. Data used in the meta-analysis, with H = habitat (1: coral reefs, 2: temperate reefs, 3: soft bottom), S: spatial scale (1: <10 km, 2: 10-100 km, 3: > 100 km), T = type of surrogate (1: higher-taxa, 2: cross-taxa, 3: subset-taxa), M = statistical method used (1: congruence in univariate biodiversity metrics, 2: congruence in multivariate biodiversity metrics, 3: representation), Tax = taxonomic level (P: phylum, C: class, O: order, F: family, G: genus, S: species), Biodiv = biodiversity metrics (incidence: incidence matrix, compos: community composition, irreplac: irreplaceability; see Table S2), H_0_ reject = whether the null hypothesis of randomness in surrogate predictions was rejected, P = probability, R² = coefficient of determination, *n* = number of samples. Note that all species surveyed were included in the analyses, except for [109-112] where species that were represented by only one or two individuals were removed.

| Test | Author | Year | H | S | T | M | Surrogate | |  |  | Target |  |  | H_0_ reject | P | R² |
| --- | --- | --- | --- | --- | --- | --- | --- | --- | --- | --- | --- | --- | --- | --- | --- | --- |
| ID |  |  |  |  |  |  |  | Group | Tax | Biodiv | Group | Tax. | Biodiv |  |  |  |
| 1 | Beger | 2003 | 1 | 2 | 2 | 1 | Coral | S | richness | Fish | S | richness | No | >0.05 | 0.084 | 36 |
| 2 | Beger | 2003 | 1 | 2 | 2 | 1 | Fish | S | richness | Fish | S | rarity | No | >0.05 | 0.078 | 36 |
| 3 | Beger | 2003 | 1 | 2 | 2 | 1 | Coral | S | richness | Coral | S | rarity | Yes | <0.01 | 0.325 | 36 |
| 4 | Beger | 2003 | 1 | 2 | 2 | 2 | Coral | S | incidence | Fish | S | incidence | No | 0.3 | 0.116 | 36 |
| 5 | Beger | 2003 | 1 | 2 | 2 | 3 | Coral | S | richness | All | S | richness | Yes | <0.05 | NA | 36 |
| 6 | Beger | 2003 | 1 | 2 | 2 | 3 | Coral | S | rarity | All | S | richness | No | >0.05 | NA | 36 |
| 7 | Beger | 2003 | 1 | 2 | 2 | 3 | Fish | S | richness | All | S | richness | Yes | <0.05 | NA | 36 |
| 8 | Beger | 2003 | 1 | 2 | 2 | 3 | Fish | S | rarity | All | S | richness | No | >0.05 | NA | 36 |
| 9 | Beger | 2007 | 1 | 2 | 2 | 2 | Fish | S | incidence | Coral | S | incidence | Yes | <0.001 | 0.518 | 29 |
| 10 | Beger | 2007 | 1 | 2 | 2 | 2 | Fish | S | incidence | Coral | S | incidence | Yes | <0.001 | 0.325 | 37 |
| 11 | Beger | 2007 | 1 | 2 | 2 | 2 | Fish | S | incidence | Coral | S | incidence | Yes | <0.001 | 0.221 | 28 |
| 12 | Beger | 2007 | 1 | 2 | 2 | 2 | Fish | S | incidence | Coral | S | incidence | Yes | <0.001 | 0.372 | 28 |
| 13 | Beger | 2007 | 1 | 2 | 2 | 2 | Fish | S | incidence | Coral | S | incidence | Yes | <0.001 | 0.533 | 45 |
| 14 | Beger | 2007 | 1 | 2 | 2 | 2 | Mollusk | S | incidence | Coral | S | incidence | No | <0.001 | 0.008 | 29 |
| 15 | Beger | 2007 | 1 | 2 | 2 | 2 | Mollusk | S | incidence | Coral | S | incidence | Yes | 0.07 | 0.032 | 37 |
| 16 | Beger | 2007 | 1 | 2 | 2 | 2 | Mollusk | S | incidence | Coral | S | incidence | Yes | <0.001 | 0.084 | 28 |
| 17 | Beger | 2007 | 1 | 2 | 2 | 2 | Mollusk | S | incidence | Coral | S | incidence | Yes | <0.001 | 0.152 | 28 |
| 18 | Beger | 2007 | 1 | 2 | 2 | 2 | Mollusk | S | incidence | Coral | S | incidence | Yes | <0.001 | 0.212 | 45 |
| 19 | Beger | 2007 | 1 | 2 | 2 | 2 | Fish | S | incidence | Mollusk | S | incidence | No | 0.09 | 0.007 | 29 |
| 20 | Beger | 2007 | 1 | 2 | 2 | 2 | Fish | S | incidence | Mollusk | S | incidence | Yes | <0.001 | 0.152 | 37 |
| 21 | Beger | 2007 | 1 | 2 | 2 | 2 | Fish | S | incidence | Mollusk | S | incidence | Yes | <0.001 | 0.096 | 28 |
| 22 | Beger | 2007 | 1 | 2 | 2 | 2 | Fish | S | incidence | Mollusk | S | incidence | Yes | <0.001 | 0.144 | 28 |
| 23 | Beger | 2007 | 1 | 2 | 2 | 2 | Fish | S | incidence | Mollusk | S | incidence | Yes | <0.001 | 0.23 | 45 |
| 24 | Beger | 2007 | 1 | 2 | 2 | 3 | Fish | S | richness | Coral | S | richness | Yes | <0.05 | NA | 28 |
| 25 | Beger | 2007 | 1 | 2 | 2 | 3 | Mollusk | S | richness | Coral | S | richness | No | >0.05 | NA | 28 |
| 26 | Beger | 2007 | 1 | 2 | 2 | 3 | Fish | S | richness | Coral | S | richness | No | >0.05 | NA | 45 |
| 27 | Beger | 2007 | 1 | 2 | 2 | 3 | Mollusk | S | richness | Coral | S | richness | Yes | <0.05 | NA | 45 |
| 28 | Beger | 2007 | 1 | 2 | 2 | 3 | Fish | S | richness | Coral | S | richness | Yes | <0.05 | NA | 29 |
| 29 | Beger | 2007 | 1 | 2 | 2 | 3 | Mollusk | S | richness | Coral | S | richness | No | >0.05 | NA | 29 |
| 30 | Beger | 2007 | 1 | 2 | 2 | 3 | Fish | S | richness | Coral | S | richness | Yes | <0.05 | NA | 37 |
| 31 | Beger | 2007 | 1 | 2 | 2 | 3 | Mollusk | S | richness | Coral | S | richness | No | >0.05 | NA | 37 |
| 32 | Beger | 2007 | 1 | 2 | 2 | 3 | Fish | S | richness | Coral | S | richness | Yes | <0.05 | NA | 28 |
| 33 | Beger | 2007 | 1 | 2 | 2 | 3 | Mollusk | S | richness | Coral | S | richness | No | >0.05 | NA | 28 |
| 34 | Beger | 2007 | 1 | 2 | 2 | 3 | Mollusk | S | richness | Fish | S | richness | No | >0.05 | NA | 28 |
| 35 | Beger | 2007 | 1 | 2 | 2 | 3 | Coral | S | richness | Fish | S | richness | No | >0.05 | NA | 28 |
| 36 | Beger | 2007 | 1 | 2 | 2 | 3 | Mollusk | S | richness | Fish | S | richness | No | >0.05 | NA | 45 |
| 37 | Beger | 2007 | 1 | 2 | 2 | 3 | Coral | S | richness | Fish | S | richness | No | >0.05 | NA | 45 |
| 38 | Beger | 2007 | 1 | 2 | 2 | 3 | Mollusk | S | richness | Fish | S | richness | Yes | <0.05 | NA | 29 |
| 39 | Beger | 2007 | 1 | 2 | 2 | 3 | Coral | S | richness | Fish | S | richness | No | >0.05 | NA | 29 |
| 40 | Beger | 2007 | 1 | 2 | 2 | 3 | Mollusk | S | richness | Fish | S | richness | No | >0.05 | NA | 37 |
| 41 | Beger | 2007 | 1 | 2 | 2 | 3 | Coral | S | richness | Fish | S | richness | No | >0.05 | NA | 37 |
| 42 | Beger | 2007 | 1 | 2 | 2 | 3 | Mollusk | S | richness | Fish | S | richness | No | >0.05 | NA | 28 |
| 43 | Beger | 2007 | 1 | 2 | 2 | 3 | Coral | S | richness | Fish | S | richness | No | >0.05 | NA | 28 |
| 44 | Beger | 2007 | 1 | 2 | 2 | 3 | Fish | S | richness | Mollusk | S | richness | No | >0.05 | NA | 28 |
| 45 | Beger | 2007 | 1 | 2 | 2 | 3 | Coral | S | richness | Mollusk | S | richness | No | >0.05 | NA | 28 |
| 46 | Beger | 2007 | 1 | 2 | 2 | 3 | Fish | S | richness | Mollusk | S | richness | No | >0.05 | NA | 45 |
| 47 | Beger | 2007 | 1 | 2 | 2 | 3 | Coral | S | richness | Mollusk | S | richness | No | >0.05 | NA | 45 |
| 48 | Beger | 2007 | 1 | 2 | 2 | 3 | Fish | S | richness | Mollusk | S | richness | Yes | <0.05 | NA | 29 |
| 49 | Beger | 2007 | 1 | 2 | 2 | 3 | Coral | S | richness | Mollusk | S | richness | No | >0.05 | NA | 29 |
| 50 | Beger | 2007 | 1 | 2 | 2 | 3 | Fish | S | richness | Mollusk | S | richness | No | >0.05 | NA | 37 |
| 51 | Beger | 2007 | 1 | 2 | 2 | 3 | Coral | S | richness | Mollusk | S | richness | No | >0.05 | NA | 37 |
| 52 | Beger | 2007 | 1 | 2 | 2 | 3 | Fish | S | richness | Mollusk | S | richness | No | >0.05 | NA | 28 |
| 53 | Beger | 2007 | 1 | 2 | 2 | 3 | Coral | S | richness | Mollusk | S | richness | No | >0.05 | NA | 28 |
| 54 | Gladstone | 2002 | 2 | 2 | 3 | 3 | Macroalgae | S | richness | All | S | richness | No | >0.05 | NA | 15 |
| 55 | Gladstone | 2002 | 2 | 2 | 3 | 3 | Mollusk | S | richness | All | S | richness | Yes | <0.05 | NA | 15 |
| 56 | Gladstone | 2002 | 2 | 2 | 3 | 1 | Mollusk | S | richness | All | S | richness | Yes | <0.01 | 0.689 | 15 |
| 57 | Gladstone | 2002 | 2 | 2 | 3 | 1 | Mollusk | S | rarity | All | S | rarity | Yes | <0.01 | 0.009 | 15 |
| 58 | Gladstone | 2002 | 2 | 2 | 3 | 1 | Macroalgae | S | richness | All | S | richness | No | >0.05 | 0.078 | 15 |
| 59 | Gladstone | 2002 | 2 | 2 | 3 | 1 | Macroalgae | S | rarity | All | S | rarity | No | >0.05 | 0.005 | 15 |
| 60 | Gladstone | 2005 | 2 | 2 | 1 | 1 | Mollusk | O | richness | Mollusk | S | richness | No | >0.05 | 0.548 | 21 |
| 61 | Gladstone | 2005 | 2 | 2 | 1 | 1 | Mollusk | F | richness | Mollusk | S | richness | Yes | <0.01 | 0.656 | 21 |
| 62 | Gladstone | 2005 | 2 | 2 | 1 | 1 | Mollusk | G | richness | Mollusk | S | richness | Yes | <0.001 | 0.922 | 21 |
| 63 | Gladstone | 2005 | 2 | 2 | 1 | 1 | Fish | O | richness | Fish | S | richness | No | >0.05 | 0.032 | 21 |
| 64 | Gladstone | 2005 | 2 | 2 | 1 | 1 | Fish | F | richness | Fish | S | richness | No | >0.05 | 0.23 | 21 |
| 65 | Gladstone | 2005 | 2 | 2 | 1 | 1 | Fish | G | richness | Fish | S | richness | Yes | <0.001 | 0.922 | 21 |
| 66 | Gladstone | 2005 | 2 | 2 | 1 | 3 | Mollusk | O | richness | Mollusk | S | richness | Yes | <0.05 | NA | 21 |
| 67 | Gladstone | 2005 | 2 | 2 | 1 | 3 | Mollusk | F | richness | Mollusk | S | richness | Yes | <0.05 | NA | 21 |
| 68 | Gladstone | 2005 | 2 | 2 | 1 | 3 | Mollusk | G | richness | Mollusk | S | richness | Yes | <0.05 | NA | 21 |
| 69 | Gladstone | 2005 | 2 | 2 | 1 | 3 | Fish | O | richness | Fish | S | richness | Yes | <0.05 | NA | 21 |
| 70 | Gladstone | 2005 | 2 | 2 | 1 | 3 | Fish | F | richness | Fish | S | richness | Yes | <0.05 | NA | 21 |
| 71 | Gladstone | 2005 | 2 | 2 | 1 | 3 | Fish | G | richness | Fish | S | richness | Yes | <0.05 | NA | 21 |
| 72 | Hirst | 2008 | 2 | 3 | 2 | 1 | Macroalgae | S | richness | Arthropod | S | richness | Yes | 0.029 | 0.084 | 57 |
| 73 | Hirst | 2008 | 2 | 3 | 2 | 1 | Macroalgae | S | biomass | Arthropod | S | richness | No | >0.05 | 0.013 | 57 |
| 74 | Hirst | 2008 | 2 | 3 | 2 | 1 | Macroalgae | S | abundance | Arthropod | S | abundance | No | >0.05 | 0.056 | 57 |
| 75 | Hirst | 2008 | 2 | 3 | 2 | 1 | Macroalgae | S | biomass | Arthropod | S | abundance | No | >0.05 | 0.041 | 57 |
| 76 | Hirst | 2008 | 2 | 3 | 2 | 2 | Macroalgae | G | compos | Arthropod | S | compos | Yes | <0.002 | 0.106 | 57 |
| 77 | Hirst | 2008 | 2 | 3 | 2 | 2 | Macroalgae | F | compos | Arthropod | S | compos | Yes | <0.002 | 0.031 | 57 |
| 78 | Hirst | 2008 | 2 | 3 | 2 | 2 | Macroalgae | O | compos | Arthropod | S | compos | No | <0.002 | 0.004 | 57 |
| 79 | Hirst | 2008 | 2 | 3 | 2 | 2 | Macroalgae | P | compos | Arthropod | S | compos | No | <0.002 | 0.001 | 57 |
| 80 | Hirst | 2008 | 2 | 3 | 2 | 2 | Macroalgae | G | compos | Arthropod | G | compos | Yes | <0.002 | 0.104 | 57 |
| 81 | Hirst | 2008 | 2 | 3 | 2 | 2 | Macroalgae | F | compos | Arthropod | G | compos | Yes | <0.002 | 0.039 | 57 |
| 82 | Hirst | 2008 | 2 | 3 | 2 | 2 | Macroalgae | O | compos | Arthropod | G | compos | No | >0.002 | 0.001 | 57 |
| 83 | Hirst | 2008 | 2 | 3 | 2 | 2 | Macroalgae | P | compos | Arthropod | G | compos | No | >0.002 | 0.001 | 57 |
| 84 | Hirst | 2008 | 2 | 3 | 2 | 2 | Macroalgae | G | compos | Arthropod | F | compos | Yes | <0.002 | 0.114 | 57 |
| 85 | Hirst | 2008 | 2 | 3 | 2 | 2 | Macroalgae | F | compos | Arthropod | F | compos | Yes | <0.002 | 0.046 | 57 |
| 86 | Hirst | 2008 | 2 | 3 | 2 | 2 | Macroalgae | O | compos | Arthropod | F | compos | No | >0.002 | 0 | 57 |
| 87 | Hirst | 2008 | 2 | 3 | 2 | 2 | Macroalgae | P | compos | Arthropod | F | compos | No | >0.002 | 0.002 | 57 |
| 88 | Hirst | 2008 | 2 | 3 | 2 | 2 | Macroalgae | G | compos | Arthropod | O | compos | Yes | <0.002 | 0.037 | 57 |
| 89 | Hirst | 2008 | 2 | 3 | 2 | 2 | Macroalgae | F | compos | Arthropod | O | compos | Yes | <0.002 | 0.03 | 57 |
| 90 | Hirst | 2008 | 2 | 3 | 2 | 2 | Macroalgae | O | compos | Arthropod | O | compos | No | >0.002 | 0.001 | 57 |
| 91 | Hirst | 2008 | 2 | 3 | 2 | 2 | Macroalgae | P | compos | Arthropod | O | compos | No | >0.002 | 0.002 | 57 |
| 92 | Hirst | 2008 | 2 | 3 | 1 | 2 | Arthropod | G | compos | Arthropod | S | compos | Yes | <0.002 | 0.916 | 57 |
| 93 | Hirst | 2008 | 2 | 3 | 1 | 2 | Arthropod | F | compos | Arthropod | S | compos | Yes | <0.002 | 0.734 | 57 |
| 94 | Hirst | 2008 | 2 | 3 | 1 | 2 | Arthropod | O | compos | Arthropod | S | compos | Yes | <0.002 | 0.271 | 57 |
| 95 | Hirst | 2008 | 2 | 3 | 1 | 2 | Arthropod | F | compos | Arthropod | G | compos | Yes | <0.002 | 0.799 | 57 |
| 96 | Hirst | 2008 | 2 | 3 | 1 | 2 | Arthropod | O | compos | Arthropod | G | compos | Yes | <0.002 | 0.3 | 57 |
| 97 | Hirst | 2008 | 2 | 3 | 1 | 2 | Arthropod | O | compos | Arthropod | F | compos | Yes | <0.002 | 0.404 | 57 |
| 98 | Hirst | 2008 | 2 | 3 | 1 | 1 | Arthropod | G | richness | Arthropod | S | richness | Yes | <0.001 | 0.96 | 57 |
| 99 | Hirst | 2008 | 2 | 3 | 1 | 1 | Arthropod | F | richness | Arthropod | S | richness | Yes | <0.002 | 0.828 | 57 |
| 100 | Hirst | 2008 | 2 | 3 | 1 | 1 | Arthropod | O | richness | Arthropod | S | richness | Yes | <0.004 | 0.144 | 57 |
| 101 | Hughes | 2002 | 1 | 3 | 2 | 1 | Coral | S | richness | Fish | S | richness | Yes | <0.05 | 0.792 | 65 |
| 102 | Hughes | 2002 | 1 | 3 | 2 | 1 | Coral | S | endemicity | Fish | S | endemicity | No | >0.05 | 0.008 | 65 |
| 103 | Karakassis | 2008 | 3 | 2 | 2 | 2 | Megafauna | S | compos | Macrofauna | S | compos | Yes | <.0001 | 0.504 | 12 |
| 104 | Karakassis | 2008 | 3 | 2 | 2 | 2 | Megafauna | S | compos | Fish | S | compos | Yes | <0.05 | 0.073 | 12 |
| 105 | Karakassis | 2008 | 3 | 2 | 2 | 2 | Megafauna | S | compos | Ciliate | S | compos | No | >0.05 | 0.006 | 12 |
| 106 | Karakassis | 2008 | 3 | 2 | 2 | 2 | Macrofauna | S | compos | Fish | S | compos | No | >0.05 | 0.048 | 12 |
| 107 | Karakassis | 2008 | 3 | 2 | 2 | 2 | Macrofauna | S | compos | Ciliate | S | compos | No | >0.05 | 0.032 | 12 |
| 108 | Karakassis | 2008 | 3 | 2 | 2 | 2 | Fish | S | compos | Ciliate | S | compos | Yes | <0.0001 | 0.462 | 12 |
| 109 | Karakassis | 2008 | 3 | 2 | 2 | 1 | Macrofauna | S | richness | Megafauna | S | richness | No | >0.05 | 0.26 | 12 |
| 110 | Karakassis | 2008 | 3 | 2 | 2 | 1 | Macrofauna | S | richness | Fish | S | richness | Yes | <0.05 | 0.49 | 12 |
| 111 | Karakassis | 2008 | 3 | 2 | 2 | 1 | Macrofauna | S | richness | Ciliate | S | richness | No | >0.05 | 0.04 | 12 |
| 112 | Karakassis | 2008 | 3 | 2 | 2 | 1 | Megafauna | S | richness | Fish | S | richness | No | >0.05 | 0.02 | 12 |
| 113 | Karakassis | 2008 | 3 | 2 | 2 | 1 | Megafauna | S | richness | Ciliate | S | richness | No | >0.05 | 0.212 | 12 |
| 114 | Karakassis | 2008 | 3 | 2 | 2 | 1 | Fish | S | richness | Ciliate | S | richness | No | >0.05 | 0.137 | 12 |
| 115 | Magierowski | 2006 | 3 | 1 | 3 | 1 | All | S | abundance | All | F | richness | Yes | <0.0001 | 0.846 | 91 |
| 116 | Magierowski | 2006 | 3 | 1 | 3 | 1 | Mollusk | F | richness | All | F | richness | Yes | <0.0001 | 0.624 | 91 |
| 117 | Magierowski | 2006 | 3 | 1 | 3 | 1 | Crustacean | F | richness | All | F | richness | Yes | <0.0001 | 0.81 | 91 |
| 118 | Magierowski | 2006 | 3 | 1 | 3 | 1 | Polychaete | F | richness | All | F | richness | Yes | <0.0001 | 0.672 | 91 |
| 119 | Magierowski | 2006 | 3 | 1 | 3 | 1 | Echinoderm | F | richness | All | F | richness | Yes | <0.0001 | 0.563 | 91 |
| 120 | Olsgard | 2000 | 3 | 3 | 1 | 1 | All | F | richness | All | S | richness | Yes | <0.05 | 0.976 | 209 |
| 121 | Olsgard | 2000 | 3 | 3 | 3 | 1 | Polychaete | S | richness | All | S | richness | Yes | <0.05 | 0.98 | 209 |
| 122 | Olsgard | 2000 | 3 | 3 | 3 | 1 | Polychaete | F | richness | All | S | richness | Yes | <0.05 | 0.96 | 209 |
| 123 | Olsgard | 2000 | 3 | 3 | 3 | 1 | Mollusk | S | richness | All | S | richness | No | >0.05 | 0.704 | 209 |
| 124 | Olsgard | 2000 | 3 | 3 | 3 | 1 | Crustacean | S | richness | All | S | richness | No | >0.05 | 0.12 | 209 |
| 125 | Olsgard | 2000 | 3 | 3 | 3 | 1 | Echinoderm | S | richness | All | S | richness | No | >0.05 | 0.049 | 209 |
| 126 | Olsgard | 2000 | 3 | 3 | 1 | 2 | All | F | compos | All | S | compos | Yes | <0.05 | 0.846 | 209 |
| 127 | Olsgard | 2000 | 3 | 3 | 3 | 2 | Polychaete | S | compos | All | S | compos | Yes | <0.05 | 0.757 | 209 |
| 128 | Olsgard | 2000 | 3 | 3 | 3 | 2 | Polychaete | F | compos | All | S | compos | No | >0.05 | 0.578 | 209 |
| 129 | Olsgard | 2000 | 3 | 3 | 3 | 2 | Mollusk | S | compos | All | S | compos | No | >0.05 | 0.49 | 209 |
| 130 | Olsgard | 2000 | 3 | 3 | 3 | 2 | Crustacean | S | compos | All | S | compos | No | >0.05 | 0.203 | 209 |
| 131 | Olsgard | 2000 | 3 | 3 | 3 | 2 | Echinoderm | S | compos | All | S | compos | No | >0.05 | NA | 209 |
| 132 | Olsgard | 2003 | 3 | 3 | 3 | 1 | Polychaete | S | richness | All | S | richness | Yes | <0.001 | NA | 152 |
| 133 | Olsgard | 2003 | 3 | 3 | 1 | 1 | Polychaete | G | richness | Polychaete | S | richness | Yes | <0.001 | 0.941 | 152 |
| 134 | Olsgard | 2003 | 3 | 3 | 1 | 1 | Polychaete | F | richness | Polychaete | S | richness | Yes | <0.001 | 0.757 | 152 |
| 135 | Olsgard | 2003 | 3 | 3 | 1 | 1 | Polychaete | O | richness | Polychaete | S | richness | Yes | <0.001 | 0.423 | 152 |
| 136 | Smith | 2005 | 2 | 2 | 3 | 2 | Mollusk | S | compos | All | S | compos | Yes | <0.05 | 0.762 | 15 |
| 137 | Smith | 2005 | 2 | 2 | 3 | 2 | Crustacean | S | compos | All | S | compos | Yes | <0.05 | 0.408 | 15 |
| 138 | Smith | 2005 | 2 | 2 | 3 | 2 | Cnidaria | S | compos | All | S | compos | Yes | <0.05 | 0.316 | 15 |
| 139 | Smith | 2005 | 2 | 2 | 3 | 2 | Gastropod | S | compos | All | S | compos | Yes | <0.05 | 0.57 | 15 |
| 140 | Smith | 2005 | 2 | 2 | 3 | 1 | Mollusk | S | richness | All | S | richness | Yes | <0.05 | 0.924 | 15 |
| 141 | Smith | 2005 | 2 | 2 | 3 | 1 | Crustacean | S | richness | All | S | richness | Yes | <0.05 | 0.805 | 15 |
| 142 | Smith | 2005 | 2 | 2 | 3 | 1 | Echinoderm | S | richness | All | S | richness | Yes | <0.05 | 0.582 | 15 |
| 143 | Smith | 2005 | 2 | 2 | 3 | 1 | Cnidaria | S | richness | All | S | richness | Yes | <0.05 | 0.493 | 15 |
| 144 | Smith | 2005 | 2 | 2 | 3 | 1 | Gastropod | S | richness | All | S | richness | Yes | <0.05 | 0.901 | 15 |
| 145 | Vanderklift | 1996 | 3 | 2 | 1 | 2 | All | G | compos | All | S | compos | Yes | <0.05 | 0.98 | 36 |
| 146 | Vanderklift | 1996 | 3 | 2 | 1 | 2 | All | F | compos | All | S | compos | Yes | <0.05 | 0.81 | 36 |
| 147 | Vanderklift | 1996 | 3 | 2 | 1 | 2 | All | O | compos | All | S | compos | Yes | <0.05 | 0.757 | 36 |
| 148 | Vanderklift | 1996 | 3 | 2 | 1 | 2 | All | C | compos | All | S | compos | No | >0.05 | 0.436 | 36 |
| 149 | Vanderklift | 1998 | 3 | 2 | 1 | 3 | All | G | incidence | All | S | incidence | Yes | <0.05 | NA | 100 |
| 150 | Vanderklift | 1998 | 3 | 2 | 1 | 3 | All | F | incidence | All | S | incidence | Yes | <0.05 | NA | 100 |
| 151 | Vanderklift | 1998 | 3 | 2 | 1 | 3 | All | C | incidence | All | S | incidence | No | >0.05 | NA | 100 |
| 152 | Vanderklift | 1998 | 3 | 2 | 1 | 3 | Plant | G | incidence | Plant | S | richness | No | >0.05 | NA | 100 |
| 153 | Vanderklift | 1998 | 3 | 2 | 1 | 3 | Plant | F | incidence | Plant | S | richness | No | >0.05 | NA | 100 |
| 154 | Vanderklift | 1998 | 3 | 2 | 1 | 3 | Plant | C | incidence | Plant | S | richness | No | >0.05 | NA | 100 |
| 155 | Vanderklift | 1998 | 3 | 2 | 1 | 3 | Invertebrate | G | incidence | Invertebrate | S | richness | Yes | <0.05 | NA | 100 |
| 156 | Vanderklift | 1998 | 3 | 2 | 1 | 3 | Invertebrate | F | incidence | Invertebrate | S | richness | Yes | <0.05 | NA | 100 |
| 157 | Vanderklift | 1998 | 3 | 2 | 1 | 3 | Invertebrate | C | incidence | Invertebrate | S | richness | Yes | <0.05 | NA | 100 |
| 158 | Vanderklift | 1998 | 3 | 2 | 1 | 3 | Fish | G | incidence | Fish | S | richness | Yes | <0.05 | NA | 100 |
| 159 | Vanderklift | 1998 | 3 | 2 | 1 | 3 | Fish | F | incidence | Fish | S | richness | Yes | <0.05 | NA | 100 |
| 160 | Ward | 1999 | 3 | 2 | 3 | 3 | Plant | S | incidence | All | S | richness | No | >0.05 | NA | 157 |
| 161 | Ward | 1999 | 3 | 2 | 3 | 3 | Fish | S | incidence | All | S | richness | Yes | <0.05 | NA | 157 |
| 162 | Ward | 1999 | 3 | 2 | 3 | 3 | Invertebrate | S | incidence | All | S | richness | Yes | <0.05 | NA | 157 |
| 163 | Terlizzi | 2009 | 3 | 2 | 1 | 2 | Mollusk | S | incidence | Mollusk | G | incidence | Yes | <0.001 | 0.968 | 45 |
| 164 | Terlizzi | 2009 | 3 | 2 | 1 | 2 | Mollusk | S | incidence | Mollusk | F | incidence | Yes | <0.001 | 0.949 | 45 |
| 165 | Terlizzi | 2009 | 3 | 2 | 1 | 2 | Mollusk | S | incidence | Mollusk | O | incidence | Yes | <0.001 | 0.468 | 45 |
| 166 | Terlizzi | 2009 | 3 | 2 | 1 | 2 | Mollusk | S | incidence | Mollusk | C | incidence | Yes | <0.001 | 0.097 | 45 |
| 167 | Terlizzi | 2009 | 3 | 2 | 1 | 2 | Mollusk | G | incidence | Mollusk | F | incidence | Yes | <0.001 | 0.98 | 45 |
| 168 | Terlizzi | 2009 | 3 | 2 | 1 | 2 | Mollusk | G | incidence | Mollusk | O | incidence | Yes | <0.001 | 0.504 | 45 |
| 169 | Terlizzi | 2009 | 3 | 2 | 1 | 2 | Mollusk | G | incidence | Mollusk | C | incidence | Yes | <0.001 | 0.104 | 45 |
| 170 | Terlizzi | 2009 | 3 | 2 | 1 | 2 | Mollusk | F | incidence | Mollusk | O | incidence | Yes | <0.001 | 0.53 | 45 |
| 171 | Terlizzi | 2009 | 3 | 2 | 1 | 2 | Mollusk | F | incidence | Mollusk | C | incidence | Yes | <0.001 | 0.115 | 45 |
| 172 | Terlizzi | 2009 | 3 | 2 | 1 | 2 | Mollusk | O | incidence | Mollusk | C | incidence | Yes | <0.001 | 0.237 | 45 |
| 173 | Terlizzi | 2009 | 3 | 2 | 1 | 2 | Mollusk | S | incidence | Mollusk | G | incidence | Yes | <0.001 | 0.874 | 45 |
| 174 | Terlizzi | 2009 | 3 | 2 | 1 | 2 | Mollusk | S | incidence | Mollusk | F | incidence | Yes | <0.001 | 0.709 | 45 |
| 175 | Terlizzi | 2009 | 3 | 2 | 1 | 2 | Mollusk | S | incidence | Mollusk | O | incidence | Yes | <0.001 | 0.241 | 45 |
| 176 | Terlizzi | 2009 | 3 | 2 | 1 | 2 | Mollusk | S | incidence | Mollusk | C | incidence | Yes | <0.001 | 0 | 45 |
| 177 | Terlizzi | 2009 | 3 | 2 | 1 | 2 | Mollusk | G | incidence | Mollusk | F | incidence | Yes | <0.001 | 0.824 | 45 |
| 178 | Terlizzi | 2009 | 3 | 2 | 1 | 2 | Mollusk | G | incidence | Mollusk | O | incidence | Yes | <0.001 | 0.272 | 45 |
| 179 | Terlizzi | 2009 | 3 | 2 | 1 | 2 | Mollusk | G | incidence | Mollusk | C | incidence | Yes | <0.001 | 0.001 | 45 |
| 180 | Terlizzi | 2009 | 3 | 2 | 1 | 2 | Mollusk | F | incidence | Mollusk | O | incidence | Yes | <0.001 | 0.432 | 45 |
| 181 | Terlizzi | 2009 | 3 | 2 | 1 | 2 | Mollusk | F | incidence | Mollusk | C | incidence | Yes | <0.001 | 0.008 | 45 |
| 182 | Terlizzi | 2009 | 3 | 2 | 1 | 2 | Mollusk | O | incidence | Mollusk | C | incidence | Yes | <0.001 | 0.143 | 45 |
| 183 | Terlizzi | 2009 | 3 | 2 | 1 | 2 | Mollusk | S | incidence | Mollusk | G | incidence | Yes | <0.001 | 0.832 | 101 |
| 184 | Terlizzi | 2009 | 3 | 2 | 1 | 2 | Mollusk | S | incidence | Mollusk | F | incidence | Yes | <0.001 | 0.743 | 101 |
| 185 | Terlizzi | 2009 | 3 | 2 | 1 | 2 | Mollusk | S | incidence | Mollusk | O | incidence | Yes | <0.001 | 0.411 | 101 |
| 186 | Terlizzi | 2009 | 3 | 2 | 1 | 2 | Mollusk | S | incidence | Mollusk | C | incidence | Yes | <0.001 | 0.294 | 101 |
| 187 | Terlizzi | 2009 | 3 | 2 | 1 | 2 | Mollusk | G | incidence | Mollusk | F | incidence | Yes | <0.001 | 0.884 | 101 |
| 188 | Terlizzi | 2009 | 3 | 2 | 1 | 2 | Mollusk | G | incidence | Mollusk | O | incidence | Yes | <0.001 | 0.458 | 101 |
| 189 | Terlizzi | 2009 | 3 | 2 | 1 | 2 | Mollusk | G | incidence | Mollusk | C | incidence | Yes | <0.001 | 0.249 | 101 |
| 190 | Terlizzi | 2009 | 3 | 2 | 1 | 2 | Mollusk | F | incidence | Mollusk | O | incidence | Yes | <0.001 | 0.548 | 101 |
| 191 | Terlizzi | 2009 | 3 | 2 | 1 | 2 | Mollusk | F | incidence | Mollusk | C | incidence | Yes | <0.001 | 0.248 | 101 |
| 192 | Terlizzi | 2009 | 3 | 2 | 1 | 2 | Mollusk | O | incidence | Mollusk | C | incidence | Yes | <0.001 | 0.294 | 101 |
| 193 | Terlizzi | 2009 | 3 | 2 | 1 | 2 | Mollusk | S | incidence | Mollusk | G | incidence | Yes | <0.001 | 0.863 | 80 |
| 194 | Terlizzi | 2009 | 3 | 2 | 1 | 2 | Mollusk | S | incidence | Mollusk | F | incidence | Yes | <0.001 | 0.674 | 80 |
| 195 | Terlizzi | 2009 | 3 | 2 | 1 | 2 | Mollusk | S | incidence | Mollusk | O | incidence | Yes | <0.001 | 0.331 | 80 |
| 196 | Terlizzi | 2009 | 3 | 2 | 1 | 2 | Mollusk | S | incidence | Mollusk | C | incidence | Yes | <0.001 | 0.072 | 80 |
| 197 | Terlizzi | 2009 | 3 | 2 | 1 | 2 | Mollusk | G | incidence | Mollusk | F | incidence | Yes | <0.001 | 0.801 | 80 |
| 198 | Terlizzi | 2009 | 3 | 2 | 1 | 2 | Mollusk | G | incidence | Mollusk | O | incidence | Yes | <0.001 | 0.329 | 80 |
| 199 | Terlizzi | 2009 | 3 | 2 | 1 | 2 | Mollusk | G | incidence | Mollusk | C | incidence | Yes | <0.001 | 0.068 | 80 |
| 200 | Terlizzi | 2009 | 3 | 2 | 1 | 2 | Mollusk | F | incidence | Mollusk | O | incidence | Yes | <0.001 | 0.403 | 80 |
| 201 | Terlizzi | 2009 | 3 | 2 | 1 | 2 | Mollusk | F | incidence | Mollusk | C | incidence | Yes | <0.001 | 0.067 | 80 |
| 202 | Terlizzi | 2009 | 3 | 2 | 1 | 2 | Mollusk | O | incidence | Mollusk | C | incidence | Yes | <0.001 | 0.089 | 80 |
| 203 | Terlizzi | 2009 | 3 | 2 | 1 | 2 | Mollusk | S | incidence | Mollusk | G | incidence | Yes | <0.001 | 0.808 | 77 |
| 204 | Terlizzi | 2009 | 3 | 2 | 1 | 2 | Mollusk | S | incidence | Mollusk | F | incidence | Yes | <0.001 | 0.778 | 77 |
| 205 | Terlizzi | 2009 | 3 | 2 | 1 | 2 | Mollusk | S | incidence | Mollusk | O | incidence | Yes | <0.001 | 0.379 | 77 |
| 206 | Terlizzi | 2009 | 3 | 2 | 1 | 2 | Mollusk | S | incidence | Mollusk | C | incidence | Yes | <0.001 | 0.066 | 77 |
| 207 | Terlizzi | 2009 | 3 | 2 | 1 | 2 | Mollusk | G | incidence | Mollusk | F | incidence | Yes | <0.001 | 0.964 | 77 |
| 208 | Terlizzi | 2009 | 3 | 2 | 1 | 2 | Mollusk | G | incidence | Mollusk | O | incidence | Yes | <0.001 | 0.486 | 77 |
| 209 | Terlizzi | 2009 | 3 | 2 | 1 | 2 | Mollusk | G | incidence | Mollusk | C | incidence | Yes | <0.001 | 0.104 | 77 |
| 210 | Terlizzi | 2009 | 3 | 2 | 1 | 2 | Mollusk | F | incidence | Mollusk | O | incidence | Yes | <0.001 | 0.49 | 77 |
| 211 | Terlizzi | 2009 | 3 | 2 | 1 | 2 | Mollusk | F | incidence | Mollusk | C | incidence | Yes | <0.001 | 0.103 | 77 |
| 212 | Terlizzi | 2009 | 3 | 2 | 1 | 2 | Mollusk | O | incidence | Mollusk | C | incidence | Yes | <0.001 | 0.204 | 77 |
| 213 | Musco | 2009 | 2 | 1 | 1 | 1 | Polychaete | G | richness | Polychaete | S | richness | Yes | <0.001 | 0.881 | 45 |
| 214 | Musco | 2009 | 2 | 1 | 1 | 1 | Polychaete | F | richness | Polychaete | S | richness | Yes | <0.001 | 0.567 | 45 |
| 215 | Tataranni | 2009 | 3 | 1 | 1 | 2 | Macrofauna | F | compos | Macrofauna | S | compos | Yes | <0.001 | 0.922 | 96 |
| 216 | Tataranni | 2009 | 3 | 1 | 1 | 2 | Macrofauna | C | compos | Macrofauna | S | compos | Yes | <0.001 | 0.319 | 96 |
| 217 | Shokri | 2009 | 3 | 1 | 2 | 1 | Annelida | S | richness | All | S | richness | Yes | <0.001 | 0.41 | 36 |
| 218 | Shokri | 2009 | 3 | 1 | 2 | 1 | Annelida | S | abundance | All | S | abundance | Yes | <0.001 | 0.26 | 36 |
| 219 | Shokri | 2009 | 3 | 1 | 2 | 1 | Arthropod | S | richness | All | S | richness | Yes | 0.002 | 0.26 | 36 |
| 220 | Shokri | 2009 | 3 | 1 | 2 | 1 | Arthropod | S | abundance | All | S | abundance | Yes | <0.001 | 0.348 | 36 |
| 221 | Shokri | 2009 | 3 | 1 | 2 | 1 | Mollusk | S | richness | All | S | richness | Yes | <0.001 | 0.26 | 36 |
| 222 | Shokri | 2009 | 3 | 1 | 2 | 1 | Mollusk | S | abundance | All | S | abundance | No | 0.16 | 0.053 | 36 |
| 223 | Shokri | 2009 | 3 | 1 | 2 | 2 | Annelida | S | incidence | All | S | incidence | Yes | <0.001 | 0.548 | 36 |
| 224 | Shokri | 2009 | 3 | 1 | 2 | 2 | Annelida | S | compos | All | S | compos | Yes | <0.001 | 0.656 | 36 |
| 225 | Shokri | 2009 | 3 | 1 | 2 | 2 | Arthropod | S | incidence | All | S | incidence | Yes | <0.001 | 0.462 | 36 |
| 226 | Shokri | 2009 | 3 | 1 | 2 | 2 | Arthropod | S | compos | All | S | compos | Yes | <0.001 | 0.548 | 36 |
| 227 | Shokri | 2009 | 3 | 1 | 2 | 2 | Mollusk | S | incidence | All | S | incidence | Yes | <0.001 | 0.548 | 36 |
| 228 | Shokri | 2009 | 3 | 1 | 2 | 2 | Mollusk | S | compos | All | S | compos | Yes | <0.001 | 0.608 | 36 |
| 229 | Shokri | 2009 | 3 | 1 | 2 | 3 | Annelida | S | richness | All | S | richness | Yes | <0.001 | NA | 36 |
| 230 | Shokri | 2009 | 3 | 1 | 2 | 3 | Arthropod | S | richness | All | S | richness | Yes | <0.001 | NA | 36 |
| 231 | Shokri | 2009 | 3 | 1 | 2 | 3 | Mollusk | S | richness | All | S | richness | Yes | <0.001 | NA | 36 |
| 232 | Shokri | 2009 | 3 | 1 | 1 | 1 | Macrofauna | G | richness | Macrofauna | S | richness | Yes | <0.001 | 0.941 | 36 |
| 233 | Shokri | 2009 | 3 | 1 | 1 | 1 | Macrofauna | F | richness | Macrofauna | S | richness | Yes | <0.001 | 0.865 | 36 |
| 234 | Shokri | 2009 | 3 | 1 | 1 | 1 | Macrofauna | O | richness | Macrofauna | S | richness | Yes | <0.001 | 0.533 | 36 |
| 235 | Shokri | 2009 | 3 | 1 | 1 | 1 | Macrofauna | C | richness | Macrofauna | S | richness | Yes | <0.001 | 0.314 | 36 |
| 236 | Shokri | 2009 | 3 | 1 | 1 | 1 | Macrofauna | P | richness | Macrofauna | S | richness | Yes | <0.001 | 0.325 | 36 |
| 237 | Shokri | 2009 | 3 | 1 | 1 | 2 | Macrofauna | G | incidence | Macrofauna | S | incidence | Yes | <0.01 | 0.941 | 36 |
| 238 | Shokri | 2009 | 3 | 1 | 1 | 2 | Macrofauna | F | incidence | Macrofauna | S | incidence | Yes | <0.01 | 0.903 | 36 |
| 239 | Shokri | 2009 | 3 | 1 | 1 | 2 | Macrofauna | O | incidence | Macrofauna | S | incidence | Yes | <0.01 | 0.64 | 36 |
| 240 | Shokri | 2009 | 3 | 1 | 1 | 2 | Macrofauna | C | incidence | Macrofauna | S | incidence | Yes | <0.01 | 0.314 | 36 |
| 241 | Shokri | 2009 | 3 | 1 | 1 | 2 | Macrofauna | P | incidence | Macrofauna | S | incidence | Yes | <0.01 | 0.292 | 36 |
| 242 | Shokri | 2009 | 3 | 1 | 1 | 2 | Macrofauna | G | compos | Macrofauna | S | compos | Yes | <0.01 | 0.98 | 36 |
| 243 | Shokri | 2009 | 3 | 1 | 1 | 2 | Macrofauna | F | compos | Macrofauna | S | compos | Yes | <0.01 | 0.941 | 36 |
| 244 | Shokri | 2009 | 3 | 1 | 1 | 2 | Macrofauna | O | compos | Macrofauna | S | compos | Yes | <0.01 | 0.774 | 36 |
| 245 | Shokri | 2009 | 3 | 1 | 1 | 2 | Macrofauna | C | compos | Macrofauna | S | compos | Yes | <0.01 | 0.563 | 36 |
| 246 | Shokri | 2009 | 3 | 1 | 1 | 2 | Macrofauna | P | compos | Macrofauna | S | compos | Yes | <0.01 | 0.533 | 36 |
| 247 | Shokri | 2009 | 3 | 1 | 1 | 3 | Macrofauna | G | irreplac | Macrofauna | S | richness | Yes | <0.001 | NA | 36 |
| 248 | Shokri | 2009 | 3 | 1 | 1 | 3 | Macrofauna | F | irreplac | Macrofauna | S | richness | Yes | <0.001 | NA | 36 |
| 249 | Shokri | 2009 | 3 | 1 | 1 | 3 | Macrofauna | O | irreplac | Macrofauna | S | richness | Yes | <0.001 | NA | 36 |
| 250 | Shokri | 2009 | 3 | 1 | 1 | 3 | Macrofauna | C | irreplac | Macrofauna | S | richness | Yes | <0.001 | NA | 36 |
| 251 | Shokri | 2009 | 3 | 1 | 1 | 3 | Macrofauna | P | irreplac | Macrofauna | S | richness | Yes | <0.001 | NA | 36 |
| 252 | Puente | 2008 | 2 | 1 | 1 | 2 | Macroalgae | F | compos | Macroalgae | S | compos | Yes | <0.001 | 0.774 | 46 |
| 253 | Puente | 2008 | 2 | 1 | 1 | 2 | Macroalgae | G | compos | Macroalgae | S | compos | Yes | <0.001 | 1 | 46 |
| 254 | Puente | 2008 | 2 | 1 | 1 | 2 | Macroalgae | F | compos | Macroalgae | S | compos | Yes | <0.001 | 1 | 46 |
| 255 | Puente | 2008 | 2 | 1 | 1 | 2 | Macroalgae | O | compos | Macroalgae | S | compos | Yes | <0.001 | 0.865 | 46 |
| 256 | Puente | 2008 | 2 | 1 | 1 | 2 | Macroalgae | G | incidence | Macroalgae | S | compos | Yes | <0.001 | 0.951 | 46 |
| 257 | Puente | 2008 | 2 | 1 | 1 | 2 | Macroalgae | F | incidence | Macroalgae | S | compos | Yes | <0.001 | 0.916 | 46 |
| 258 | Puente | 2008 | 2 | 1 | 1 | 2 | Macroalgae | O | incidence | Macroalgae | S | compos | Yes | <0.001 | 0.483 | 46 |
| 259 | Puente | 2008 | 2 | 1 | 1 | 2 | Macroalgae | G | compos | Macroalgae | S | compos | Yes | <0.001 | 1 | 46 |
| 260 | Puente | 2008 | 2 | 1 | 1 | 2 | Macroalgae | F | compos | Macroalgae | S | compos | Yes | <0.001 | 1 | 46 |
| 261 | Puente | 2008 | 2 | 1 | 1 | 2 | Macroalgae | O | compos | Macroalgae | S | compos | Yes | <0.001 | 1 | 46 |
| 262 | Puente | 2008 | 2 | 1 | 1 | 2 | Macroalgae | G | incidence | Macroalgae | S | compos | Yes | <0.001 | 0.612 | 46 |
| 263 | Puente | 2008 | 2 | 1 | 1 | 2 | Macroalgae | F | incidence | Macroalgae | S | compos | Yes | <0.001 | 0.408 | 46 |
| 264 | Puente | 2008 | 2 | 1 | 1 | 2 | Macroalgae | O | incidence | Macroalgae | S | compos | Yes | <0.001 | 0.359 | 46 |
