# Supplementary material for: Effectiveness of Biological Surrogates for Predicting Patterns of Marine Biodiversity: A Global Meta-Analysis
Source: PLoS One. 2011 Jun 14;6(6):e20141. doi: 10.1371/journal.pone.0020141 (PMC3114784; doi:10.1371/journal.pone.0020141)
Supplement: Text S1 — WinBUGS code for Bayesian modelling. (DOC) [file pone.0020141.s003.doc]

**Effectiveness of Biological Surrogates for Predicting Patterns of Marine Biodiversity : a Global Meta-Analysis**

Mellin et al. – Supporting Information

**Text S1** WinBUGS code for Bayesian modelling

Example WinBUGS code for fitting Bayesian hierarchical model of surrogate effectiveness (defined as the probability that surrogate predictions are non-random) with informative priors. This example illustrates (1) a model with logit link and binomial variance

model {

# Prior for random effect for(j in 1:J2){

rID[j] ~ dnorm(0, tau.rID)

}

# Dummy covariates

for (i in 1:J){

levelA[i] <- equals(covariate[i], 1)

levelB[i] <- equals(covariate[i], 2)

levelC[i] <- equals(covariate[i], 3)

}

# Logit model with binomial variance

for (i in 1:J){

y[i] ~ dbern(p[i])

logit(p[i]) <- beta0 * levelA[i] + beta1 * levelB[i] + beta2 * levelC[i] + rID[study[i]]

}

# Priors

beta0 ~ dnorm(0, 0.001)

beta1 ~ dnorm(0, 0.001)

beta2 ~ dnorm(0, 0.001)

# Hyperprior for random effect precision

tau.rID ~ dgamma(0.001, 0.001)

sigma.rID <- 1/tau.rID

}

and (2) a model with logit link and beta variance

model {

# Prior for random effect for(j in 1:J2){

rID[j] ~ dnorm(0, tau.rID)

}

# Dummy covariates

for (i in 1:J){

levelA[i] <- equals(covariate[i], 1)

levelB[i] <- equals(covariate[i], 2)

levelC[i] <- equals(covariate[i], 3)

}

# Logit model with beta variance

for (i in 1:J){

y[i] ~ dbeta(a[i], b[i])

a[i] <- mu[i] * gamma

b[i] <- (1 - mu[i]) * gamma

logit(mu[i]) <- beta0 * levelA[i] + beta1 * levelB[i] + beta2 * levelC[i] + rID[study[i]] }

# Priors

beta0 ~ dnorm(0, 0.001)

beta1 ~ dnorm(0, 0.001)

beta2 ~ dnorm(0, 0.001)

gamma ~ dgamma(0.01, 0.01)

# Hyperprior for random effect precision

tau.rID ~ dgamma(0.001, 0.001)

sigma.rID <- 1/tau.rID

}
