# Supplementary material for: Effectiveness of Biological Surrogates for Predicting Patterns of Marine Biodiversity: A Global Meta-Analysis
Source: PLoS One. 2011 Jun 14;6(6):e20141. doi: 10.1371/journal.pone.0020141 (PMC3114784; doi:10.1371/journal.pone.0020141)
Supplement: Text S2 — Literature reviewed. (DOC) [file pone.0020141.s004.doc]

**Effectiveness of Biological Surrogates for Predicting Patterns of Marine Biodiversity : a Global Meta-Analysis**

Mellin et al. – Supporting Information

**Text S2** Literature reviewed.

Beger M., G. P. Jones, and P. L. Munday. 2003. Conservation of coral reef biodiversity: a comparison of reserve selection procedures for corals and fishes. Biological Conservation **111:**53-62.

Beger M., S. A. McKenna, and H. P. Possingham. 2007. Effectiveness of surrogate taxa in the design of coral reef reserve systems in the Indo-Pacific. Conservation Biology **21:**1584-1593.

Gladstone W. 2002. The potential value of indicator groups in the selection of marine reserves. Biological Conservation **104:**211-220.

Gladstone W. and T. Alexander. 2005. A test of the higher-taxon approach in the identification of candidate sites for marine reserves. Biodiversity and Conservation **14:**3151-3168.

Hirst A. J. 2008. Surrogate measures for assessing cryptic faunal biodiversity on macroalgal-dominated subtidal reefs. Biological Conservation **141:**211-220.

Hughes T. P., D. R. Bellwood, and S. R. Connolly. 2002. Biodiversity hotspots, centres of endemicity, and the conservation of coral reefs. Ecology Letters **5:**775-784.

Karakassis I., A. Machias, P. Pitta et al. 2006. Cross-community congruence of patterns in a marine ecosystem: Do the parts reflect the whole? Marine Ecology Progress Series **310:**47-54.

Magierowski R. H. and C. R. Johnson. 2006. Robustness of surrogates of biodiversity in marine benthic communities. Ecological Applications **16:**2264-2275.

Musco L., A. Terlizzi, M. Licciano, and A. Giangrande. 2009. Taxonomic structure and the effectiveness of surrogates in environmental monitoring: a lesson from polychaetes. Marine Ecology-Progress Series **383:**199-210.

Olsgard F., T. Brattegard, and T. Holthe. 2003. Polychaetes as surrogates for marine biodiversity: lower taxonomic resolution and indicator groups. Biodiversity and Conservation **12:**1033-1049.

Olsgard F. and P. J. Somerfield. 2000. Surrogates in marine benthic investigations: Which taxonomic unit to target? Journal of Aquatic Ecosystem Stress and Recovery **7:**25-42.

Puente A. and J. A. Juanes. 2008. Testing taxonomic resolution, data transformation and selection of species for monitoring macroalgae communities. Estuarine Coastal and Shelf Science **78:**327-340.

Shokri M. and W. Gladstone. 2009. Higher taxa are effective surrogates for species in the selection of conservation reserves in estuaries. Aquatic Conservation: Marine and Freshwater Ecosystems **19:**626-636.

Shokri M. R., W. Gladstone, and A. Kepert. 2009. Annelids, arthropods or molluscs are suitable as surrogate taxa for selecting conservation reserves in estuaries. Biodiversity and Conservation **18:**1117-1130.

Smith S. D. A. 2005. Rapid assessment of invertebrate biodiversity on rocky shores: where there's a whelk there's a way. Biodiversity and Conservation **14:**3565-3576.

Tataranni M., F. Maltagliati, A. Floris, A. Castelli, and C. Lardicci. 2009. Variance estimate and taxonomic resolution: An analysis of macrobenthic spatial patterns at different scales in a Western Mediterranean coastal lagoon. Marine Environmental Research **67:**219-229.

Terlizzi A., M. J. Anderson, S. Bevilacqua et al. 2009. Beta diversity and taxonomic sufficiency: Do higher-level taxa reflect heterogeneity in species composition? Diversity and Distributions **15:**450-458.

Vanderklift M. A., T. J. Ward, and C. A. Jacoby. 1996. Effect of reducing taxonomic resolution on ordinations to detect pollution-induced gradients in macrobenthic infaunal assemblages. Marine Ecology Progress Series **136:**137-145.

Vanderklift M. A., T. J. Ward, and J. C. Phillips. 1998. Use of assemblages derived from different taxonomic levels to select areas for conserving marine biodiversity. Biological Conservation **86:**307-315.

Ward T. J., M. A. Vanderklift, A. O. Nicholls, and R. A. Kenchington. 1999. Selecting marine reserves using habitats and species assemblages as surrogates for biological diversity. Ecological Applications **9:**691-698.
